# Anti-Butterfly Effect in Ribavirin Studied by Combined Experiment (PXRD/^1^H-^14^N NQR Cross-Relaxation Spectroscopy), Quantum Chemical Calculations, Molecular Docking, Molecular Dynamics Simulations, and Novel Structure-Binding Strength and Quadrupolar Indices

**DOI:** 10.3390/molecules30051096

**Published:** 2025-02-27

**Authors:** Jolanta Natalia Latosińska, Magdalena Latosińska, Janez Seliger, Veselko Žagar, Tomaž Apih

**Affiliations:** 1 Faculty of Physics and Astronomy, Adam Mickiewicz University, Uniwersytetu Poznańskiego 2, 61-614 Poznań, Poland; magdalena.latosinska@amu.edu.pl; 2 Faculty of Mathematics and Physics, University of Ljubljana, Jadranska 19, 1000 Ljubljana, Slovenia; janez.seliger@fmf.uni-lj.si; 3 “Jožef Stefan” Institute, Jamova 39, 1000 Ljubljana, Slovenia; veselko.zagar@ijs.si (V.Ž.); tomaz.apih@ijs.si (T.A.)

**Keywords:** ribavirin, anti-viral, ^1^H-^14^N NQR cross-relaxation spectroscopy, structure-binding affinity, density functional theory, molecular dynamic simulations, anti-butterfly effect, structure-binding indices, quadrupolar indices

## Abstract

Ribavirin, 1-(β-D-Ribofuranosyl)-1*H*-1,2,4-triazole-3-carboxamide, which is included in the list of drugs recommended in the guidelines for the diagnosis and treatment of SARS-CoV-2 infection, has been the subject of experimental and theoretical investigation. The most thermodynamically stable polymorphic form was studied using ^1^H-^14^N NQR cross-relaxation, periodic DFT/QTAIM/RDS/3D Hirshfeld surfaces, and molecular docking. For the first time, a ^1^H-^14^N cross-relaxation spectrum of ribavirin was recorded and interpreted. Twelve resonance frequencies were assigned to four inequivalent nitrogen positions in the molecule using combined experimental techniques and solid-state quantum chemical calculations. The influence of the structural alteration on the NQR parameters was modeled using GGA/RPBE. The differences in the binding pattern of ribavirin, acadesine, inosine, guanosine, and favipiravir-ribofuranosyl in the solid state and the protein-ligand complex were assessed to elucidate the differences in the binding mechanism at the molecular level due to aglycone modification. The replacement of the carbon adjacent to the ribose with nitrogen, in conjunction with the absence of oxygen at the 2-position of the ring, resulted in an increased flexibility of the RBV structure in comparison to the favipiravir-ribofuranosyl structure. The present study identified the intramolecular hydrogen bond NH···N in RBV as playing a crucial role in the formation of a quasi-five-membered ring. However, this bond was proven to be too weak to force positioning of the amide group in the ring plane. The ribofuranosyl in RBV inhibits tautomerism and freezes the conformation of the amide group. The results of the molecular dynamics simulations demonstrated that RBV and favipiravir-ribofuranosyl incorporated into the RNA primer exhibited comparable stability within the protein binding region. The titular anti-butterfly (inverted butterfly) effect is associated with the consequences of both the changes in aglycone moiety and the neighborhood alteration. Seven structure-binding strength indices and six novel quadrupolar indices defined in this study have been proven to facilitate the evaluation of the similarity of binding motifs in the solid state and protein-ligand complex.

## 1. Introduction

In a previous publication [1], we examined the impact of a minor local structural alteration on biological activity. We have shown that a slight structural alteration (the inversion of the hydroxyl group at the 2′-position of the glycone) leads to a change in the binding affinity and, as a consequence, alters the biological activity from anti-depressant and DNA/RNA block builder (cytidine) to powerful anti-cancer (cytarabine). The present paper discusses the inverse effect, whereby biological activity remains nearly unchanged despite significant modification in the molecular structure and binding pattern.

Our research focuses on ribavirin (1-(*β*-D-Ribofuranosyl)-1*H*-1,2,4-triazole-3-carboxamide, RBV; CAS: 36791-04-5), a synthetic nucleoside discovered by the International Chemical & Nuclear Corporation (now Valeant Pharmaceuticals) under the National Cancer Institute’s Virus-Cancer program and patented in 1972 [2,3]. RBV is included in the WHO Model Lists of Essential Medicines based on public health relevance, evidence of efficacy and safety, and comparative cost-effectiveness [4].

It has a formal status of a generic, thanks to which it can be offered not only by the original but also alternative manufacturers (trade names: Copegus (Roche, Basel, Switzerland), Rebetol (Schering-Plough Brussels, Belgium, Merck Sharp & Dohme Ltd., Rahway, US and Fulford India Ltd., Mumbai, India), Ribavirin (Biopartners, Los Angeles, CA, USA; Teva Pharms/TEVA, Tel-Aviv, Israel; Three Rivers Global Pharma, London UK; Aurobindo Pharma Ltd., Hyderabad, India; Sandoz, Zydus Pharms, Pennington, NJ, USA), Ibavyr (Pendopharm/Pharmascience Inc., Montreal, QC, Canada), Virazole (Valeant Pharm. Intl. Inc./Bausch Health Companies Inc., Laval, QC, Canada), Rebetol (Merck Sharp & Dohme Ltd., Rahway, NJ, US), Ribasphere (Three Rivers Pharms, Cranberry Township, PA, USA), Moderiba (AbbVie Co., North Chicago, IL, USA), Dormant (Pendopharm Division of Pharmascienco Inc., Montreal, QC, Canada), Rebretron (Schering-Plough Canada Inc., Kirkland, QC, Canada), Victrelis Triple (Merck Canada Inc., QC, Canada), Pegasys RNV (Hoffmann-La Roche Ltd., Basel, Switzerland), Pegetron (Merck Canada Inc., Quebeck, QC, Canada), and Ribavin/Virazide (Lupin Laboratories Ltd./Pinnacle, Mumbai, India).

In 1986, RBV was approved by the U.S. Food and Drug Administration (FDA) for the treatment of chronic hepatitis C viral infections (HCV) in combination with pegylated interferon alfa (INF-a) [5,6]. It is currently used in combination with interferon alfa-2a or alfa-2b and with peginterferon alfa-2a or alfa-2b, as RBV monotherapy has proven ineffective. Presently, the original role of RBV in HCV therapy has diminished, but it is still recommended in combined treatments with selected interferon-free, direct-acting antivirals, such as sofosbuvir [7]. RBV has also recently been used in clinical trials in treating other types of hepatitis: A, B, and E [8,9,10].

Furthermore, it is a broad-spectrum antiviral agent used against a wide variety of human viral infections. RBV inhibits the replication of RNA and DNA of myxoviruses, paramyxoviruses, arenaviruses, retroviruses, flaviviruses, and poxviruses. It could be effective in the early stages of viral hemorrhagic fevers including Lassa and Venezuelan hemorrhagic fevers (*Arenaviridae* viruses), Crimean-Congo hemorrhagic fever (*Orthonairovirus*), Nipah (*Henipavirus*), Sin Nombre (*Hantavirus*) and flaviviruses (*Orthoflavivirus*) infections, human immunodeficiency virus (*HIV*, *Lentivirus*), and human adenoviruses (HAdVs, *Adenoviridae*) [11]. Moreover, RBV is used as an inhaled medication for severe respiratory syncytial virus (*RSV*) infections in children. Preliminary data suggest that RBV may be effective against herpes simplex viruses (*HSV*-1 and *HSV-2*) and canine distemper [12]. However, there has been evidence of poor in vitro and in vivo activity against selected filoviruses (Ebola and Marburg) and flaviviruses (Dengue, yellow fever, Omsk hemorrhagic fever, and Kyasanur forest disease). According to the ClinicalTrials.gov database, several clinical trials have been conducted on the effectiveness of RBV in combination with favipiravir, IFN-beta, nitrazoxamide, or corticosteroids against COVID-19. Recently, the COVID-19 guidelines for diagnosis and treatment incorporated RBV in the list of recommended drugs.

In addition, RBV has been demonstrated to exert anti-proliferative and cytotoxic effects on ovarian cancer OVCAR-5 [13]. At present, RBV shows promise in the treatment of specific types of cancers with elevated levels of the eukaryotic translation initiation factor (eIF4E). eIF4E is a potent oncogene, elevated in 50–100% of approximately 30% of human cancers (e.g., Hodgkin and non-Hodgkin lymphomas, chronic myelogenous leukemia (CML), and subtypes of acute myeloid leukemia (AML), as well as cancers of the breast, prostate, lung, head, and neck (including glioblastoma), and colon (especially those that are human papillomaviruses (HPV)-related)). RBV and its active metabolite ribavirin triphosphate (RTP) bind eIF4E in the region of the m^7^G cap-binding site [14,15] and thus inhibit the activity of this oncogene. Additionally, RBV has been demonstrated to diminish the expression of vascular endothelial growth factor (VEGF), thereby impeding tumor growth, peritoneal permeability, and ascites formation in hepatocellular carcinoma [16].

RBV has attracted interest for more than four decades due to its distinctive range of activities and the lack of clarity surrounding its mechanism of action [17].

A deeper understanding of the mechanism of action of ribavirin stimulates renewed interest in its potential as pharmaceutical agent for the treatment of cancer as well as infections caused by highly mutated forms of SARS-CoV-2. Nevertheless, the adverse effects of RBV, including systemic toxicity (e.g., hemolytic anemia), an increased risk of pruritus, rash, cough, neuropsychiatric side effects (e.g., insomnia), and tetragenicity restrict its use [18]. It is therefore evident that understanding the action of ribavirin, which facilitates the search for new analogues with reduced toxicity and increased antiviral activity, remains an important area of research.

The exact mechanism of action of RBV has not been fully elucidated. It is believed that RBV exerts its activity through five distinct mechanisms, two of which are indirect (inosine monophosphate dehydrogenase inhibition, immunomodulatory effects) and three of which are direct (interference with RNA capping, polymerase inhibition, lethal mutagenesis). It functions as a prodrug, exerting antiviral activity through its active phosphorylated metabolite ribavirin 5-triphosphate (RTP) and also possibly through ribavirin 5-monophosphate (RMP) [19,20]. This metabolic activation occurs intracellularly by various kinases, including adenosine kinase [21]. Its metabolite, RTP, is an inhibitor of inosine monophosphate (IMP) dehydrogenase, an enzyme that catalyzes the nicotinamide adenine dinucleotide (NAD+)-dependent oxidation of inosine monophosphate (IMP) to xanthosine monophosphate (XMP). Inosine-5′-monophosphate dehydrogenase (IMPDH) expression has been found to be upregulated in some tumor tissues and cell lines, making it a drug target for immunosuppressive and cancer chemotherapy. The experimental evidence also supports different mechanisms of action. RBV can inhibit purine biosynthesis at the level of IMP dehydrogenase and as a precursor to the di- and triphosphates, which inhibit viral RNA-dependent RNA polymerases. The RTP blocks viral RNA synthesis and viral mRNA capping. Thus, ribavirin, similarly to favipiravir, has indirect (immunomodulatory) and direct (RNA mutagen) antiviral activities [11].

**Figure 1:**
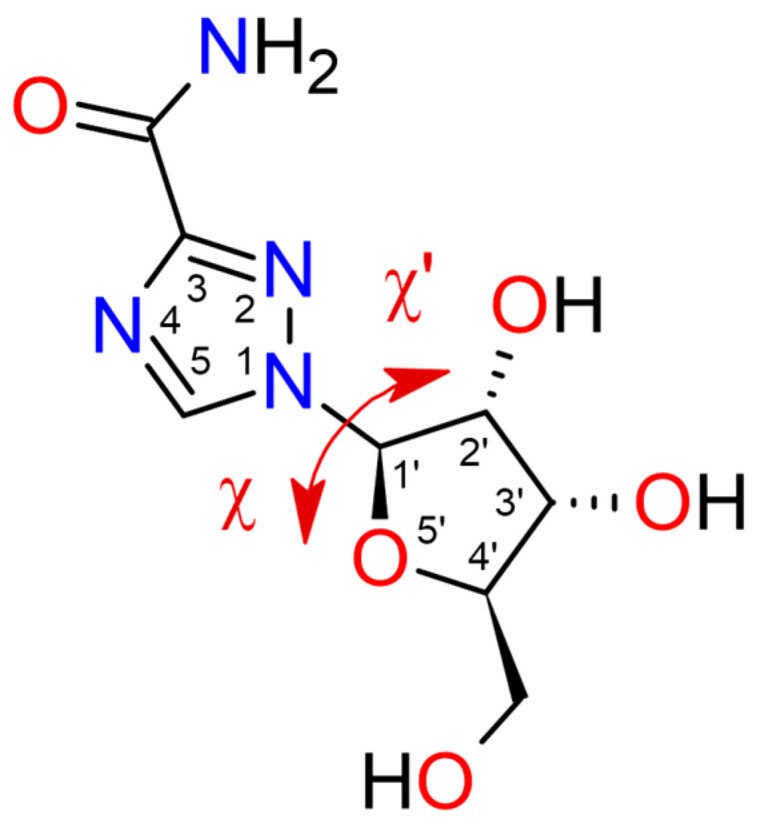
Molecular structure of nucleoside with a sugar group (glycone) bonded to a non-sugar (aglycone) via glycone-N-aglycone bridge (β-N1-glycosidic bond); χ—the rotation angle C(5)N(1)CO; and χ′—the rotation angle N(2)N(1)CO, both indicated by the red arrow.

Structurally, ribavirin, as shown in Figure 1, combines a rigid aglycone (triazole moiety) with a glycone (ribofuranosyl). The C(1) of a ribosyl moiety is N-linked to the 1,2,4-triazole ring via β-N1-glycosidic bond, which enables conformational changes that facilitate the formation of polymorphic and amorphous phases.

**Figure 2:**
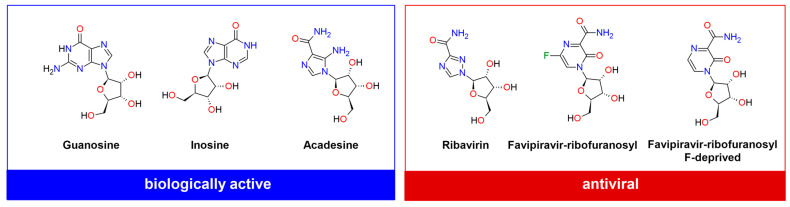
Structural formula of guanosine, inosine, acadesine (5-aminoimidazole-4-carboxamide-1-β-d-ribofuranoside, AICA-riboside/AICAR), ribavirin (1-(β-D-Ribofuranosyl)-1*H*-1,2,4-triazole-3-carboxamide, RBV), favipiravir-ribofuranosyl (6-fluoro-3-hydroxy-2-pyrazinecarboxamide-ribofuranosyl), and favipiravir-ribofuranosyl F-deprived (3-hydroxy-2-pyrazinecarboxamide-ribofuranosyl).

The ribofuranosyl fragment confers the ability to incorporate into RNA and DNA and thus represents a common component of a class of pharmaceuticals with established anti-viral and anti-cancer properties. The triazole ring is a common pharmacophoric feature of drugs used in the treatment of fungal infections, as well as other conditions such as CCR5-tropic HIV-1 infections or breast cancers. A basic component of the aglycone, 1,2,4-triazole, acts as an antifungal, while 3-amino-1,2,4-triazole is an herbicide effective against weeds [22]. Its presence facilitates the coordination of nitrogen atoms –N= with the iron of heme, as well as π-π binding with tryptophan. Furthermore, it inhibits various enzymes by binding key or critical C residues in the active site. Both aglycone with an amide group and glycone increase the number of degrees of freedom of RBV as well as its ability to form hydrogen bonds, which promotes polymorphism. It is noteworthy that, from a structural perspective, RBV bears resemblance to guanosine (required for an RNA splicing reaction in mRNA; a neuroprotective agent reducing neuroinflammation, oxidative stress, and excitotoxicity), inosine (regulates RNA editing, metabolic enzyme activity, and signaling pathways, promoting T cell proliferation and differentiation and enhancing response to immune-checkpoint inhibitors therapy), acadesine (hypoglycemic, cardioprotective and antineoplastic agent), and favipiravir-ribofuranosyl and its F-deprived analogue (both antiviral) (see Figure 2 and Table 1).

Ribavirin, favipiravir-ribofuranosyl, and acadesine contain a carboxamide-modified nucleobase, which is thought to be the determining factor for antiviral activity, but only the first two exhibit this kind of activity. Of the last two, only favipiravir-ribofuranosyl is an antiviral broad-spectrum drug. A thorough examination of favipiravir and its ribofuranosyl form has been conducted in our previous papers [23,24].

Despite the promising medical applications of RBV, there is a scarcity of research on its chemical properties. Hence, this study employs a combination of experimental (^1^H-^14^N nuclear quadrupole resonance cross-relaxation spectroscopy [25,26], powder X-ray diffraction (PXRD) [27]) as well as theoretical (modern density functional theory (DFT), Bader’s quantum theory of atoms in molecules (QTAIM) [28,29], reduced density gradient (RDS) [30], and 3D Hirshfeld surface-based [31,32]) approaches.

In chemistry and pharmaceuticals, ^14^N NQR spectroscopy is a particularly valuable experimental technique [1,23,33,34,35,36,37,38]. One of its main advantages is that the resonance lines are not broadened by the random orientations of powder grains like in NMR. Instead of relying on an external magnetic field to induce energy level splitting, NQR makes use of the nucleus’s intrinsic properties: the electric quadrupole moment of the nitrogen interacts with the electric field gradient (EFG) produced by the local electron density distribution, which is specific to each compound. As a result, the energy level splittings are strongly influenced by the chemical bonding of the quadrupole atom, which gives rise to distinctive spectra for different chemical compounds. Additionally, the splittings are modified by the position of other charges in the same molecule, as well as the relative positions of the surrounding molecules in the crystal.

In contrast to NMR, where the resonance frequency is determined mainly by the external magnetic field magnitude and then shifted by local environment interactions, in NQR, the changes in the electron density distribution occurring in the local environment modify the main interaction directly. Therefore, the NQR parameters represent an excellent criterion for the validation of wave functions in quantum chemical calculations. The electric field gradient (EFG) tensor, defined as the set of the second-order derivatives of the electrostatic potential, is very sensitive even to minor changes in the electron density distribution at the nucleus. It is a measure of the inhomogeneity of the external electric field at the nucleus site and as a first-order molecular property can be calculated using the wave function. Conversely, the accuracy of the reproduction of the EFG by means of the wave function renders it possible to evaluate its quality [39]. The enhanced reproducibility of its value is indicative of a more reliable wave function, which in turn provides deeper insight into the three-dimensional crystalline packing at the atomic and molecular levels.

Moreover, ^14^N NQR combined with an in-depth computational study of the interaction pattern can help to predict the ability of the molecule to bind to specific biological targets and thus assess its pharmaceutical attractiveness [1,23,34]. The triazole heterocyclic ring, amide functional group, and ribofuranosyl moiety facilitate RBV involvement in a variety of intermolecular interactions, including dispersive (van der Waals and steric) and electrostatic (hydrogen bond) interactions. Their competition/interplay is of paramount importance for efficient binding in the solid state and protein-ligand complex. Analysis of the interactions in the RBV crystals permits drawing conclusions on both the character and paths of connections between a given RBV as a ligand with SARS-CoV-2 RNA-dependent RNA polymerase (RdRp) to make a protein–ligand complex inhibiting SARS-CoV-2 synthesis. Insight into the interactions that are necessary for efficient recognition and binding of RBV molecules provides guidance for the synthesis of novel active pharmaceutical ingredients.

## 2. Results and Discussion

### 2.1. Experimental Investigation

#### 2.1.1. Polymorph Evaluation by PXRD

**Figure 3:**
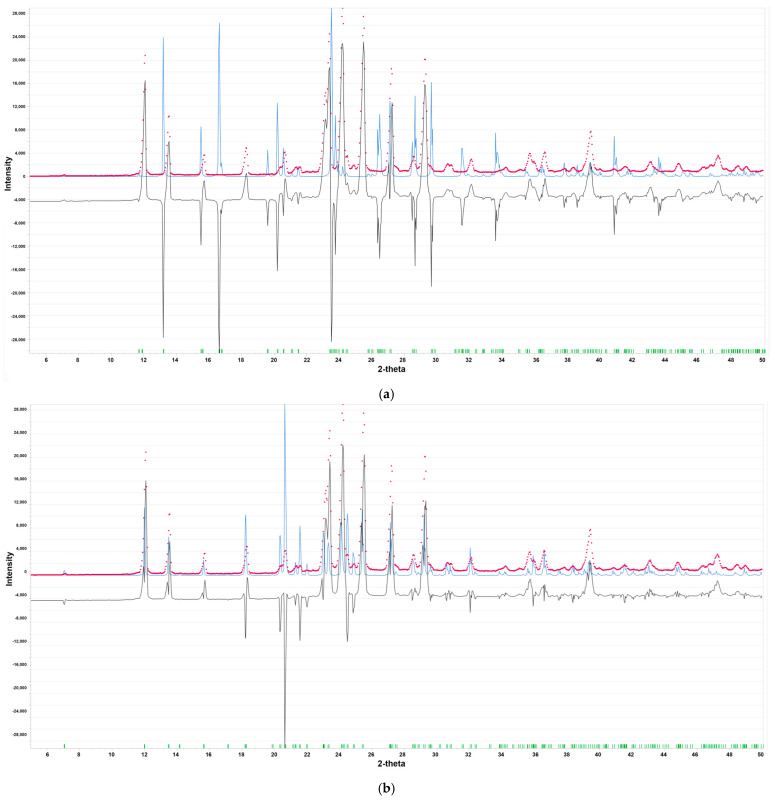
The powder X-ray diffraction pattern of (**a**) form I and (**b**) form II. The PXRD of the commercial RBV is in red; the PXRD pattern calculated under the assumption of the known X-ray structures (reference) is in blue; and the difference between experimental data and reference is in black; the observed reflections are depicted in green. The characteristic peaks at 7.1°, 12.0°, 13.5°, 15.6°, and 18.2° (form II) are clearly visible.

The presence of polymorphs can adversely affect the performance and toxicity of the active pharmaceutical ingredient (API). Therefore, the quality of the raw material was verified through the utilization of X-ray diffraction (PXRD). PXRD enables the detection and quantification of polymorphic composition by exploiting distinct powder diffraction patterns that provide unique crystal structures’ fingerprints. Based on the PXRD pattern, the sample under investigation was found to contain stable form II of RBV (Figure 3).

The peaks at 7.1°, 12.0°, 13.5°, 15.6°, and 18.2° are the characteristic features of this stable form [40]. The disparities between the PXRD patterns for the commercial RBV sample and both forms are evident. Both forms, I and II, crystallize in space group 2_1_2,_1_2_1_ with a = 14.863(8) Å, b = 7.512(2) Å, and c = 8.788(1) Å, and a = 25.034(8) Å, b = 7.719(2) Å, and c = 5.289(1) Å, respectively [41]. Form II differs from form I in its ribosyl conformation, whereas the aglycone part exhibits structural conservation between the two forms. The environment of the nitrogen nuclei located in the conserved aglycone moiety thus seems to be, at the first glance, analogous in both forms.

#### 2.1.2. ^1^H-^14^N NQR Cross-Relaxation Spectroscopy

The ^14^N NQR line positions were indirectly measured by slightly modified versions of ^14^N-^1^H cross-relaxation (CR) spectroscopy experiments.

The ^1^H-^14^N double resonance technique used in this study is a combination of the cross-relaxation technique [25,42,43] and a new cross-polarization technique designed particularly for the fast field-cycling (FFC) relaxometers. (An analysis of the new technique will be published elsewhere.) The cross-relaxation technique consists of proton polarization in a high magnetic field, magnetic flux density reduction to a low value B for a time τ, and an increase of the magnetic field to the value at which the proton NMR signal is measured. By scanning the low magnetic flux density B in the range where the proton NMR frequency may match the ^14^N NQR frequencies, we observe a dip in the proton NMR signal intensity when the proton NMR frequency matches a ^14^N NQR frequency. The strength of this dip strongly depends on the ^14^N spin-lattice relaxation rate. When it is high, the dip is strong. The strength of this dip does not depend on the ^14^N NQR frequency. The main disadvantages of using this technique with a FFC relaxometer are high power consumption and inadequate cooling of the main coil, since the duration of the proton polarization in the high magnetic field must be equal to several proton spin-lattice relaxation times T_1_.

The new technique consists of a switch on a low magnetic field with the flux density B for a time τ and then an increase of the magnetic field flux density to a high value at which the proton NMR signal is measured. When the proton NMR frequency in the low magnetic field matches a ^14^N NQR frequency, then the first ^14^N partially increases proton magnetization. Due to the Boltzmann population of the ^14^N quadrupole energy levels, the two ^14^N quadrupole energy levels involved in this process are unequally populated. The two proton energy levels are, immediately after the switching on of the low magnetic field, still equally populated. When an equilibrium in the two-spin system is established, the proton spin system gains some magnetization. This equilibrium is established in a time shorter than 1 ms, so the time τ may be rather short. By increasing the time τ, the proton magnetization relaxes toward its equilibrium value in the low magnetic field B. When, by chance, the ^14^N spin-lattice relaxation rate is high, its contribution to the proton spin-lattice relaxation may be large, and the proton magnetization will reach its equilibrium value in a shorter time than in the case when the two spin systems are not coupled. By scanning the low magnetic flux density B in the range where the proton NMR frequency may match the ^14^N NQR frequencies, we observe a peak in the proton NMR signal intensity when the proton NMR frequency matches a ^14^N NQR frequency. In contrast to the cross-relaxation technique, these peaks are stronger at high ^14^N NQR frequencies and weaker at low ^14^N NQR frequencies.

**Figure 4:**
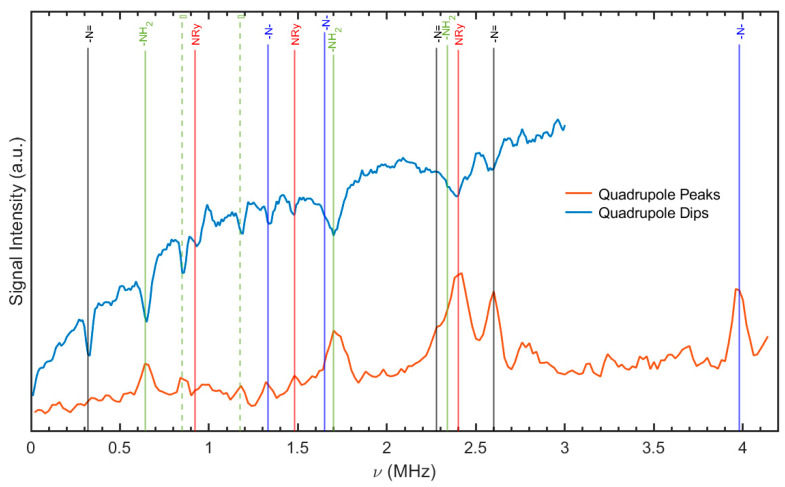
The quadrupole peaks and dips in the experimentally determined ^14^N NQR spectrum in ribavirin, RBV at 295 K. The provisional assignment of the ^1^H-^14^N NQR cross-relaxation spectrum in RBV. The dashed vertical lines indicate the double quantum transitions.

The ^1^H-^14^N NQR cross-relaxation spectrum and position of the lines for RBV are shown in Figure 4. The frequencies as well as the quadrupole coupling constant, e^2^qQ/h, and the asymmetry parameter, η, are calculated using Equations (1) and (2):

(1) $$\left|\frac{e^{2}Qq}{h}\right|=\frac{2}{3}\left(\nu_{+}+\nu_{-}\right)$$

(2) $$\eta =\frac{3\left(\nu_{+}-\nu_{-}\right)}{\nu_{+}+\nu_{-}}$$

Results are collected in Table 2.

The lack of multiplicity in the NQR spectrum suggests the existence of equivalent molecules per unit cell, which is in accordance with the X-ray data [41]. However, as demonstrated in Figure 3, the NQR spectrum offers substantiating evidence for the molecular instability of the -NH_2_ and ribofuranosyl groups, attributable to molecular motions. The supplementary peaks are denoted by a dashed line. One set of NQR frequencies for each nitrogen is very convenient, but interpreting a spectrum consisting of 12 lines is still not an easy task, even though in each set of 3 resonance lines, one frequency depends on two others. The chemical non-equivalence of the four nitrogen nuclei in the RBV molecule is attributable to the distinctive characteristics of its surrounding environment. These features encompass hybridization, substituent effects, conformation, and binding involvement, all of which play a role in shaping the molecule’s electron density distribution. Some guidance can be obtained by comparison with structural analogues. So far, only a few compounds structurally similar to RBV have been investigated, and the basic aglycone building block, 1*H*-1,2,4-triazole, seem to be the closest (Table 3).

The frequencies of the –NH_2_ group can be distinguished from others due to the strong proton–nitrogen interaction. The assignment of the remaining frequencies, especially from both –N=, presented a challenge. Both –N= type nitrogen sites in the RBV have sp^2^ hybridization and π electrons delocalized over the ring. In the absence of participation in hydrogen bonds, the quadrupole coupling constants and asymmetry parameters will undergo minimal alteration because of the solid-state effect. The nitrogen atom is engaged in two N–C covalent bonds, exhibiting a high value of the asymmetry parameter that is comparable to that observed for the –N= nitrogen atom in cytidine or cytosine [1]. Detection of the resonance lines from them requires advanced fast field-cycling (FFC) relaxometry techniques. In the case of nitrogen >N(1), which forms the glycone-N-aglycone bridge, a low asymmetrical parameter would be anticipated, similarly to cytarabine [1]. This assumption is contingent on the premise that the molecule participates in three N-C covalent bonds. The low value of the asymmetry parameter of the EFG tensor is a defining feature in this case. The neighboring –N(2)= atom binding N(1), however, introduces a bond-symmetry-breaking effect, which leads to an increased value of the asymmetry parameter. The frequencies for this site could be detected using the multiple frequency sweeping and dual-frequency irradiation techniques. Based on the experimental data, it seems that the two sets of resonance lines yielding the lowest η should be assigned to the –N(4)= and –NH_2_ sites, and the others to the two remaining –N=. The provisional individual site assignments provided in Table 2 are made under the above-mentioned assumptions.

### 2.2. Solid-State DFT Calculations

The utilization of quantum chemical calculations in the modelling of NQR spectra enables the verification of the accuracy of the frequency assignment proposed above. The efficacy of this technique is most apparent in scenarios characterized by relatively wide resonance lines, those which overlap significantly or are numerous. The accessibility of X-ray structural data facilitates examination of the two distinct conformations and the surrounding environment of the molecule within the crystal structure.

The preceding optimization of the positions of the hydrogen atoms in the –NH_2_ group is of great importance, as the NQR parameters of this group are highly sensitive to the length of the NH bond; thus, it was imperative to ascertain the optimal configuration. The partial geometry optimization concerning the hydrogen atoms’ positions was performed at the GGA/RBPE level of the theory under the assumption of the RBV crystalline structure, forms I and II.

In the next step, a full geometry optimization of the solid-state structures was performed. For the fixed-cell optimization, which facilitates the refinement of the geometry of a 3D periodic system, the same GGA/RPBE functional was applied. This approach enables the straightforward rectification of discrepancies that arise during Rietveld refinement or that are a consequence of thermal vibrations.

**Figure 5:**
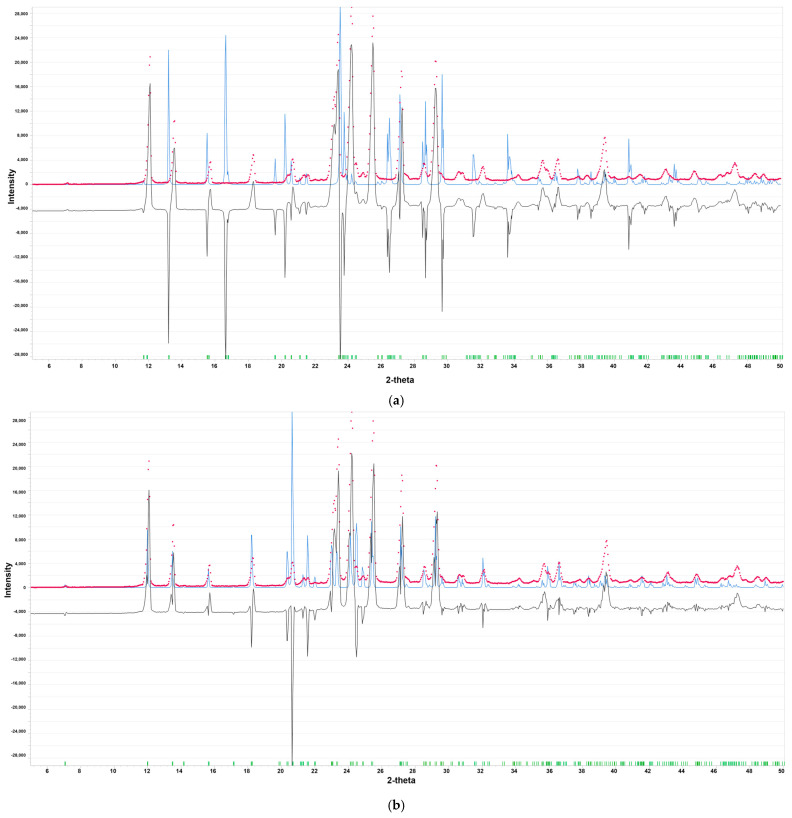
The PXRD pattern of (**a**) form I and (**b**) form II. The plot for the fully optimized structures, the experimental data, and the difference between experiment and simulation are depicted in blue, red, and black, respectively, the observed reflections are depicted in green.

The simulated PXRD spectra for the fully optimized structures of both forms I and II are shown in Figure 5. It is evident that even full geometry optimization exerts a negligible influence on the PXRD pattern. There is a substantial discrepancy between forms I and II (i.e., the full geometry optimization retained the individual features of both forms). Furthermore, the original X-ray structures [41] demonstrate a high level of refinement. The full optimization of the structures results in only minor alterations to the patterns.

**Figure 6:**
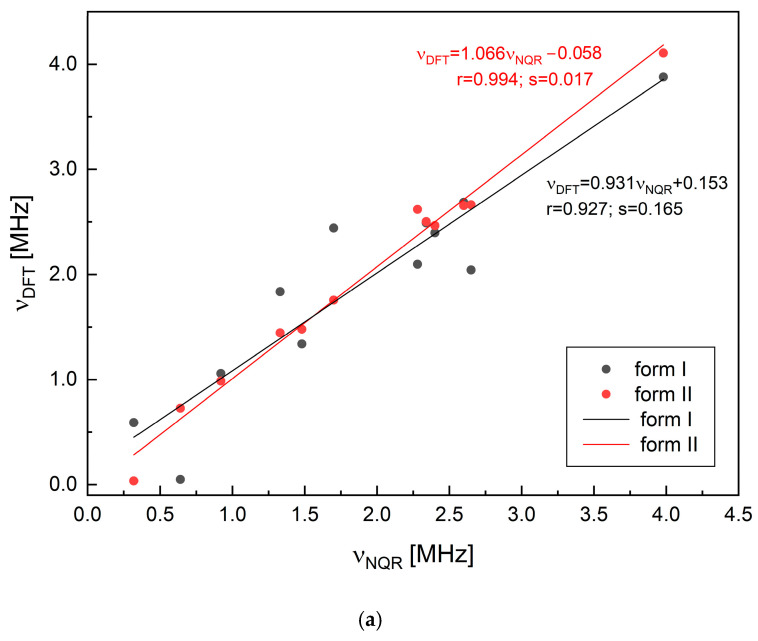

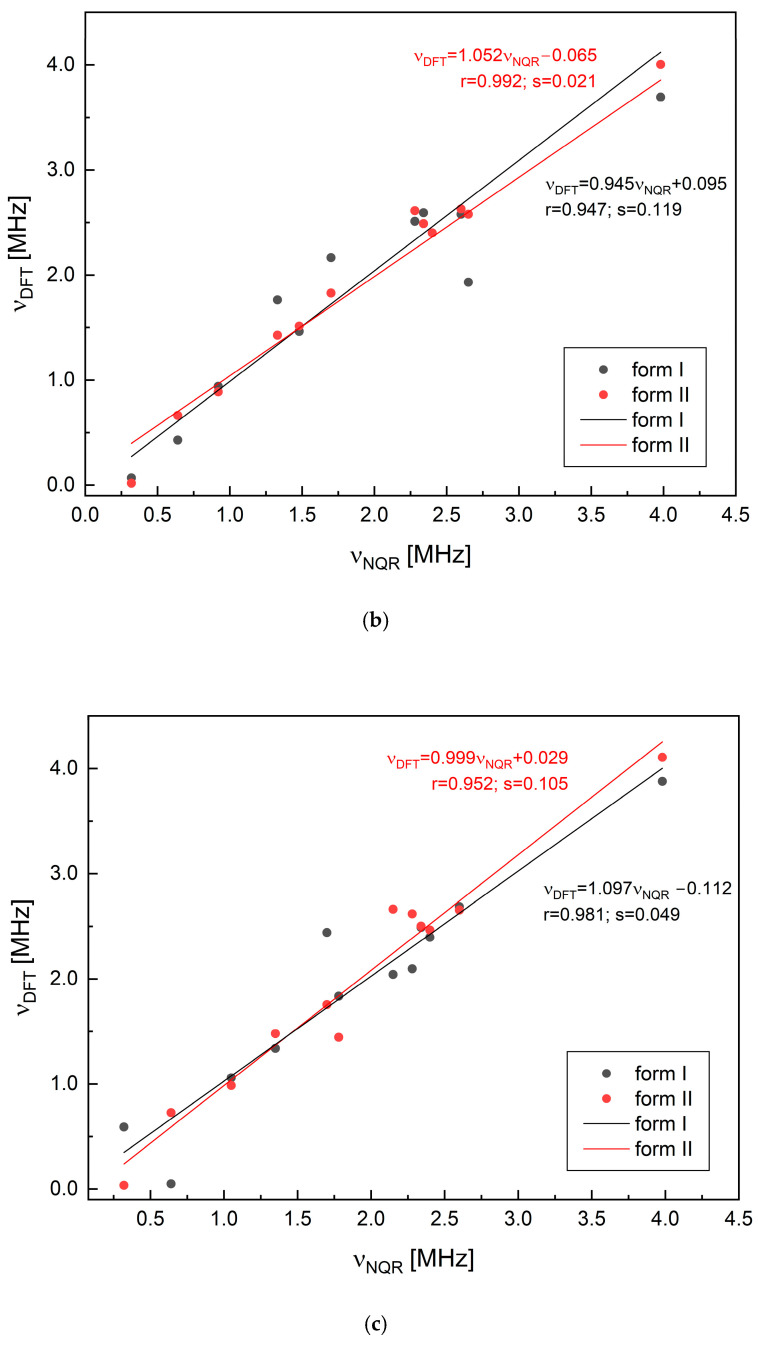
The correlation between the experimental and calculated (GGA/RPBE level, solid) ^14^N frequencies for RBV: (**a**) assignment shown in Table 2 (hydrogen positions optimized), (**b**) assignment shown in Table 2 (full geometry optimization), and (**c**) alternative assignment (r—Pearson’s correlation coefficient, s—curve fit standard deviation).

The NQR parameters and resonance frequencies calculated at the GGA/RPBE level of the theory are listed in Table 4 and visualized in Figure 6.

Despite the high correlation coefficients observed in both cases (0.993 for form II and 0.923 for form I), the dispersion is markedly higher when form I is assumed. The alternative assignment of frequencies for –N(2)= and >NRy is less reliable despite the reasonable correlation coefficients (0.981 for form II and 0.954 for form I); the points scattering is much higher. The full geometry optimization does not enhance the correlation between the calculated and experimental NQR frequencies. The discrepancy between the correlation coefficients is a bit higher. In terms of the EFG and bond strength analysis, the optimization of hydrogen positions is of greater significance. As demonstrated, the positioning of heavy atoms within a well-refined structure does not necessitate optimization. However, the comparison of the NQR parameters listed in Table 4 confirm that NQR spectra constitute a highly sensitive tool for the structural differences.

It is well known that the results of DFT calculations depend strongly on the choice of the exchange–correlation functional. The choice of RBPE leads to a more reliable reproduction of the EFG components than the alternative GGA functionals [1]. The discrepancy between the experimental result and theoretical prediction is diminished. The cluster-based approach, an alternative to solid-state calculations, was originally proposed by us for cytosine [47] and subsequently validated. Since it is not always feasible to perform calculations for 3D-periodic structures or to obtain stable structures (true minimum of the potential energy surface), even in cases where the convergence criteria are tightened, this original technique was proposed. It facilitates the reconstruction of the intermolecular interactions from the nearest neighborhood of the molecule. The calculations performed for six-molecule clusters at the M062X/6-311+G(d,p) level of theory confirmed the correctness of the frequency assignment. The correlation coefficients and curve fit standard deviations were 0.826 and 0.855, and 0.46 and 0.33, for form I and form II, respectively.

Nevertheless, the NQR spectrum, similarly to PXRD, proves that the tested specimen contains form II. Furthermore, it suggests that the environment of the nitrogen nuclei located in the conserved aglycone moiety is highly different in both forms. In general, combined experimental and theoretical NQR data suggest that –NH_2_ and to a lesser extent –N= are involved in hydrogen bonds in solid state. The amine –NH_2_ group is flexible, has lone pairs localized on the atoms adjacent to the π system, and is prone to forming strong N–H···O or N–H···N hydrogen bonds. The low value of the asymmetry parameter for –N= (ring position N(4)) indicates that this nitrogen atom is involved in intramolecular hydrogen bonding, which may be subject to modulation by proton transfer. A comparison of the NQR parameters for RBV and 1*H*-1,2,4-triazole reveals an increase in e^2^Qq/h and a decrease in η at NH_2_, and an increase in both e^2^Qq/h and η, upon ribofuranosyl and carboxamide substitution.

### 2.3. Binding Motifs in the Solid State

#### 2.3.1. 3D Hirshfeld Surfaces

According to the Etter rules, originally implied by Donohue [48,49], the molecule will attempt to form as many hydrogen bonds (HBs) as possible. The presence of the four donors (amide and hydroxide) and acceptors (i.e., two-aromatic-ring nitrogen atoms and the oxygens of amide and ribose) in the RBV molecule makes it highly susceptible to the formation of HBs. The feasibility of some HBs (which are further reinforced by van der Waals, dispersion, or stacking interactions) is constrained by the specific conformation of RBV in the solid or protein–ligand complex. The modification concerns solely the conformation of the ribofuranosyl moiety with respect to the aglycone, thereby facilitating spatial packing. The 1,2,4-triazole moiety in RBV exists in only one tautomeric form, 1*H*-1,2,4-triazole, because the interconversion to 4*H*-1,2,4-triazole is locked by the glycone moiety (Figure 1). Furthermore, according X-ray data, the conformation of the carboxamide substituent at C(3) is fixed upon polymorphic transition. These observations, akin to those made regarding the NQR parameters, indicate the potential existence of an intramolecular bond, which increases the rigidity of the 1*H*-1,2,4-triazole-3-caboxylamide and generates an additional quasi-ring. The use of quantum chemical calculations can facilitate a greater understanding of this phenomenon.

A comparison of the values of the conformation angle, <C(5)N(1)CO, listed in Table 5, reveals that ribavirin in the stable form adopts a conformation most similar to inosine (stable form) or guanosine (hydrated form), but different from that of the unstable forms of inosine or ribavirin, as well as T-1106. In addition, the analysis suggests that fluorine forces a specific conformation of ribofuranosyl in T-705, thereby confirming our earlier assumptions [23].

In order of decreasing conformation angle, the compounds under investigation can be ordered in the following way:

Inosine, β-form > ribavirin, form II > guanosine > acadesine > T-705 > inosine, α-form > ribavirin, form I > T-1106 (3)

**Figure 7:**
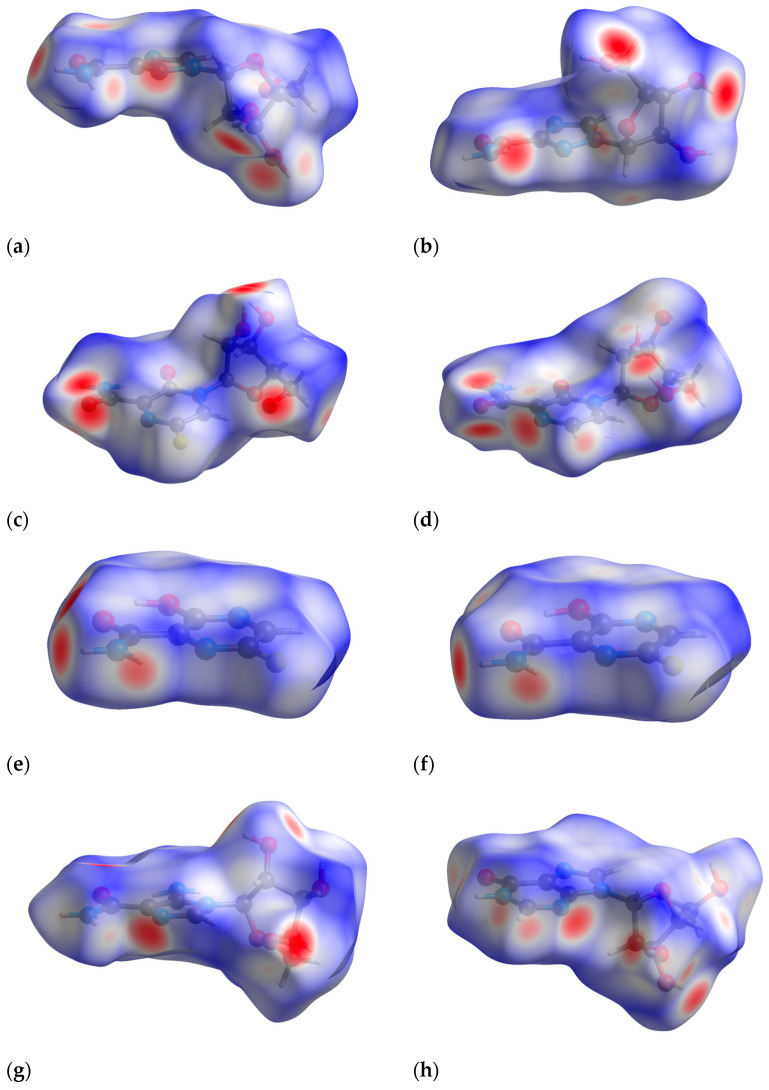
The intermolecular interactions visualized in the d_norm_ surface projected over 3D Hirshfeld surfaces in a red-white-blue scheme (red: negative, white: zero, blue: positive), with red representing small-range contacts such as hydrogen bonds, white representing contacts of approximately van der Waals radii, and blue representing the remaining, considerably longer, connections: (**a**) RBV form I (3D HS: volume = 239.87 Å^3^, area = 239.84 Å^2^, globularity = 0.778, asphericity = 0.173), (**b**) RBV form II (3D HS: volume = 249.87 Å^3^, area = 242.07 Å^2^, globularity = 0.793, asphericity = 0.138), (**c**) T-705 (3D HS: volume = 297.25 Å^3^, area = 271.71 Å^2^, globularity = 0.793, asphericity = 0.155), (**d**) T-1106 (3D HS: volume = 274.84 Å^3^, area = 258.29 Å^2^, globularity = 0.791, asphericity = 0.163), (**e**) favipiravir form I 296K (3D HS: volume = 153.78 Å^3^, area = 162.04 Å^2^, globularity = 0.857, asphericity = 0.115), (**f**) favipiravir form II 298K (3D HS: volume = 153.77 Å^3^, area = 162.34 Å^2^, globularity = 0.855, asphericity = 0.116), (**g**) acadesine (3D HS: volume = 271.02 Å^3^, area = 255.00 Å^2^, globularity = 0.794, asphericity = 0.183), and (**h**) inosine β-form (3D HS: volume = 270.20 Å^3^, area = 261.64 Å^2^, globularity = 0.773, asphericity = 0.163).

To ascertain the impact of the phenomenon on the formation of bonds, we applied the 3D Hirshfeld surfaces (3D HS), associated with 2D molecular fingerprints (2D FP), and enrichment ratios (ER) approaches. The topological properties of 3D HS, curvature, curvedness, shape index, distances from the surface to the nearest external (d_e_) and internal (d_i_) core, percentages of the surface corresponding to different types of contacts, and enrichment ratios reveal important packing features. In Figure 5, the 3D HS are depicted with the d_norm_ surface projected over them, along with a representation of the topological parameters for each of them. The red spots on the 3D HS (d_norm_) depict the strongest interactions between the neighboring molecules (Figure 6) The strength of the bond is shown by the intensity of the red color. (The blue regions can be considered as contacts deprived). The quantitative measures (total volume, area, globularity, and asphericity) of the 3D HS (Figure 5) offer some insight into their properties. The higher globularity for RBV II, T-705, and T-1106 promote isotropic behavior by the enhancement of force distribution in the crystal lattice, which affects both bond lengths and flexibility. The dissimilarities in asphericity between both RBV forms indicate disparate bond length stability, thereby enabling us to posit that the structural responses to changes in external conditions will not be especially flexible. The difference in <C(5)N(1)CO angle between T-105 and RBV form II is −45.87°, while that between T-105 and form I is −154.48°. The conformation of RBV form II is therefore closer to T-105 and T-1106 than that of RBV form I, which is reflected by the values of the topological parameters (Figure 7).

**Figure 8:**
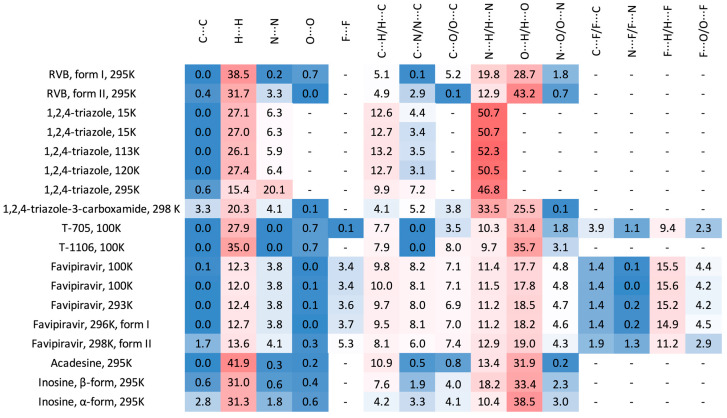
Heatmap visualizing the percentage contributions to the 3D Hirshfeld surface calculated for each pair of species. (The red-white-blue scheme, with dark red indicating high percentages and dark blue indicating very low percentages, was used. The source of X-Ray data [41,50,51,52,53,55,56,57,58,59,60,61,62]; temperature corresponds to the X-ray study).

The heatmap, which offers a visual representation of the percentage contributions of the individual contacts to the 3D HS, is shown in Figure 8.

The percentage contributions to the 3D Hirshfeld surface remained nearly unaltered by the variation in temperature, apart from the low-quality 1*H*-1,2,4-triazole structure. As much as 9–51%, 12–38%, and 17–43% of the percentage contributions comes from N···H/H···N, H···H, O···H/H···O contacts, respectively. The C···H/H···C contributions are within the range of 4.9–13.2%. The F···H/H···F contributions are within the range from 9 to 15.6%. The remaining homonuclear (C···C, N···N and O···O) or heteronuclear (N···O/O···N, N···C/C···N and C···O/O···C) contributions are negligibly small. The O···H/H···O-type contacts are the most prevalent in form II of RBV, whereas in form I, the H···H-type contacts are the most prominent. In RBV (form II), favipiravir and its active form (T-705), the same contact type predominates, i.e., O···H/H···O > H···H > N···H/H···N. The presence of fluoride in favipiravir and T-705 does not affect this order, but results in a reduction in the percentage contributions from H···H and an increase in the percentage contributions of F···H/H···F. Regarding 1*H*-1,2,4-triazole-3-carboxamide, the sequence of the percentage contributions from contacts can be delineated as follows: N···H/H···N > O···H/H···O > H···H. It is noteworthy that the N···H/H···N and H···H contacts assume particular significance, which can be attributed to the enhanced presence of nitrogen atoms. The presence of a carboxamide group and fluorine in both favipiravir and 1*H*-1,2,4-triazole-3-carboxamide results in a shift in the percentage contributions from N···H/H···N, O···H/H···O, and (to a lesser extent) H···H to F···H/H···F. The disparity in the proportion of atoms contributing to the 3D HS between the two RBV forms is approximately 41.6%, a considerable difference when compared to the 1.3% variation observed between the favipiravir forms. This finding is somewhat unexpected, given that, based on the differences in crystal symmetry, a more pronounced shift in favipiravir would be anticipated. Nevertheless, these findings are consistent with the significant similarity observed in the quantitative measures of the 3D HS in favipiravir polymorphs in comparison to those of RBV.

Following ribofuranosyl substitution, the percentage contribution of N···H/H···N contacts exhibit a marginal increase. In contrast, the replacement of 6-fluoro-3-hydroxy-2-pyrazine-carboxamide with 1*H*-1,2,4-triazole-3-carboxylamide results in a twofold increase in value. Subsequent ribofuranosyl substitution and 6-fluoro-3-hydroxy-2-pyrazine-carboxamide replacement with 1*H*-1,2,4-triazole-3-carboxylamide results in an increase in the percentage contributions of H···H contacts.

The Euclidean distance (ED) and root mean square deviation (RMSD) are the global differences between the percentage contributions of all contacts (Table 6).

As is evident from the ED or RMSD, form II of RBV is most similar in terms of its interaction pattern to T-1106, followed by T-705, RBV form I, and 1*H*-1,2,4-triazole-3-carboxamide.

#### 2.3.2. Enrichment Ratios

The enrichment ratios, E_XY_, derived from the decomposition of the crystal contact surface between pairs of interacting chemical species, are listed in Table 7. They offer some additional insight into the intermolecular contacts between each pair of the atomic species, X and Y, which can be privileged (E_XY_ > 1), neutral (E_XY_ = 1), or disfavored (E_XY_ < 1).

The percentages of the molecular surfaces among the studied crystalline structures differ slightly, by about 3–10%. Nevertheless, the percentages of the molecular surface for inosine and T-1106 are close to that observed for form II of RBV. The Euclidean distance (ED) between the enrichment ratios in RBV form I and the studied compounds (Table 8) takes the lowest values for T-1106, followed by favipiravir, acadesine, and inosine (α-form).

Consequently, the privileged and disfavored contacts between every two atomic species in RBV form II are more similar to those in inosine and T-1106 than form I of RBV. The balance between N···H/H···N and O···H/H···O contacts in form II of RBV, T-705, and T-1106 is shifted to the former, while in the other studied compounds it is to the latter. The enrichment ratios for form I of RBV are similar to those for T-1106 and inosine α, while those for form II of RBV are similar to those for inosine form β and favipiravir.

The binding pattern exhibited by the crystal structure of acadesine is relatively divergent from that observed in the structure of form II of RBV, despite the notable similarity between the molecules of acadesine and RBV. The inosine molecule, despite its comparatively reduced similarity to RBV compared to acadesine, forms a network of crystalline contacts that more closely resemble those observed in RBV than in acadesine.

#### 2.3.3. 2D Molecular Fingerprints 

**Figure 9:**
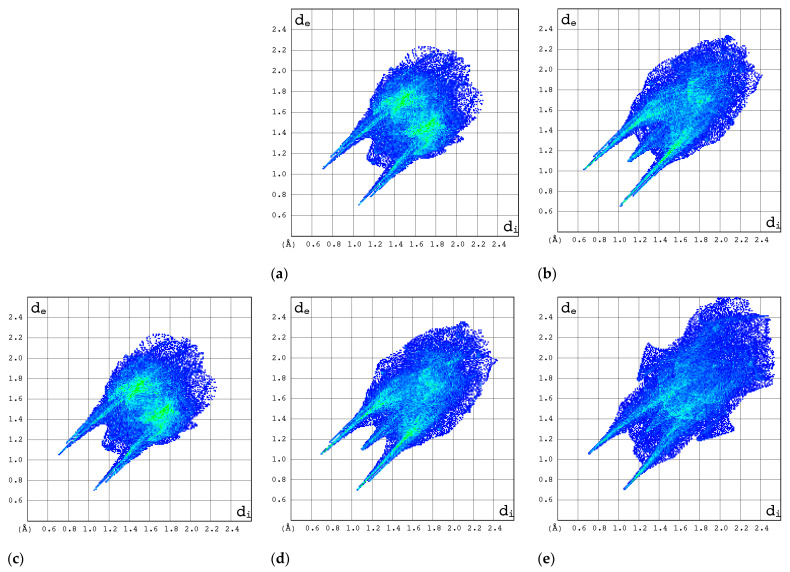

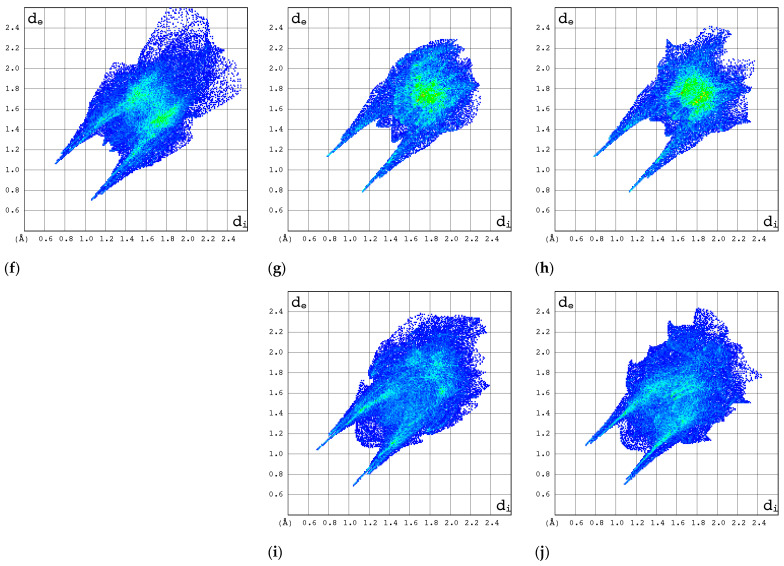
The intermolecular interactions visualized in the 2D FP (d_i_/d_e_) plot (the chromaticity of a given hue is expressed on a scale of blue to red, with higher values indicating greater intensity): (**a**) RBV form I, fully optimized geometry; (**b**) RBV form II, fully optimized geometry; (**c**) RBV form I; (**d**) RBV form II; (**e**) T-705; (**f**) T-1106; (**g**) favipiravir, form I; (**h**) favipiravir, form II; (**i**) inosine; and (**j**) acadesine.

The contributions of the heavy atoms (C, N, O, or F) to the hydrogen bonds or strong intermolecular contacts can be identified by means of 2D molecular fingerprints (2D FPs) (i.e., d_e_/d_i_ plots). As can be seen from Figure 8, the 2D FP plots differ significantly in shape and size for the compounds under investigation. Full optimization of the geometry of both RBV forms has a marginal effect on the interaction pattern as revealed by 2D FP (Figure 9a–d). In general, the 2D FPs of RBV form I resemble those of favipiravir forms I and II. Furthermore, the 2D FP of the form II of RBV is close to those of T-705 and T-1106. The cloud of points in RBV form II is found to be both thinner and more scattered in comparison to that of RBV form I and favipiravir forms I and II. Nevertheless, this phenomenon manifests to a lesser extent in comparison to that observed in T-705 and T-1106.

**Figure 10:**
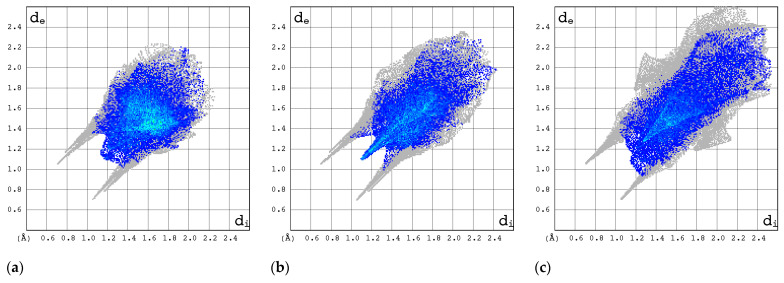

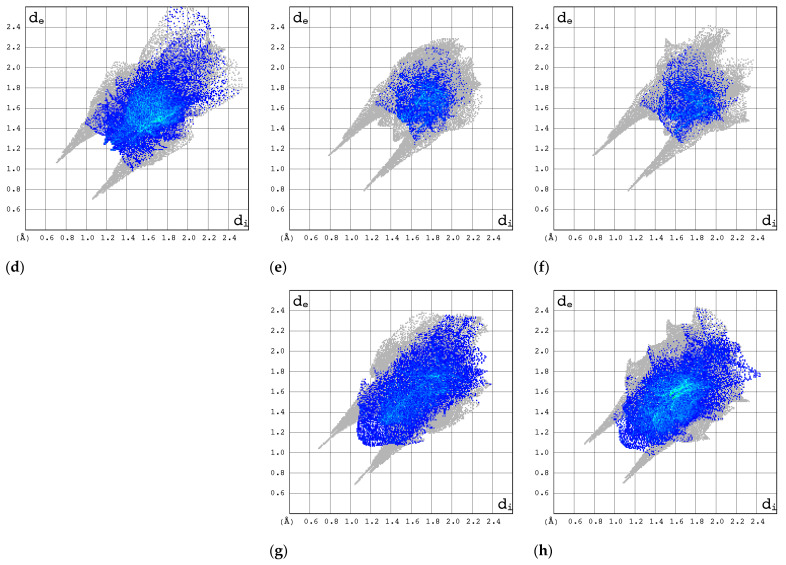
The H···H contacts visualized in the 2D FP (d_i/_d_e_) plot (the chromaticity of a given hue is expressed on a scale of blue to red, with higher values indicating greater intensity; the boundaries of the full imprint for the entire molecule are shown in gray): (**a**) RBV form I; (**b**) RBV form II; (**c**) T-705; (**d**) T-1106; (**e**) favipiravir, form I; (**f**), favipiravir, form II; (**g**) inosine; and (**h**) acadesine.

**Figure 11:**
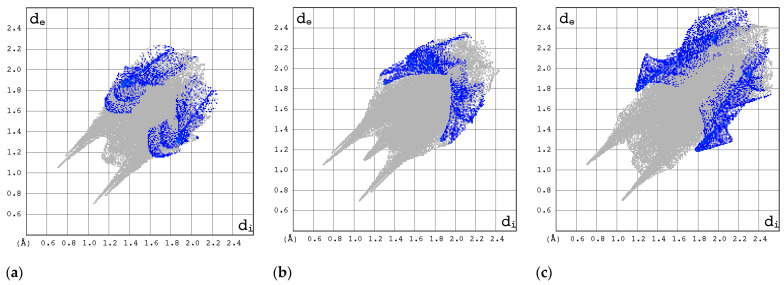

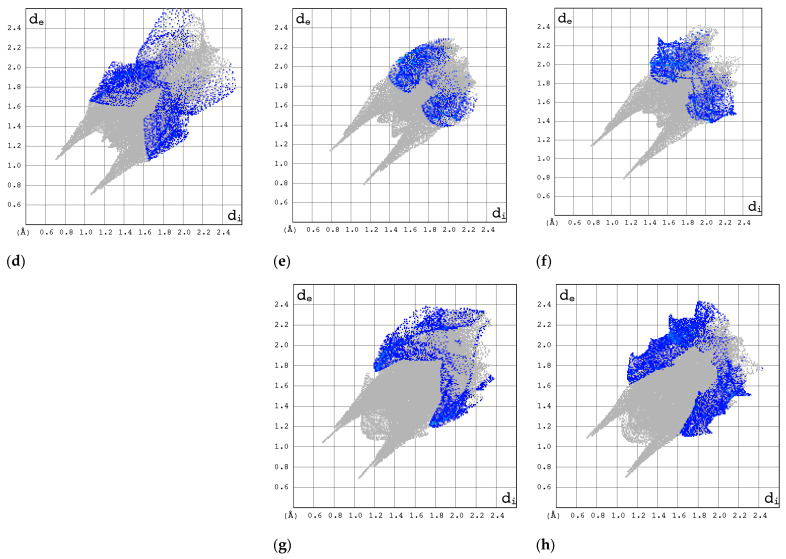
The C···H/H···C contacts visualized in the 2D FP (d_i/_d_e_) plot (the chromaticity of a given hue is expressed on a scale of blue to red, with higher values indicating greater intensity; the boundaries of the full imprint for the entire molecule are shown in gray): (**a**) RBV form I; (**b**) RBV form II; (**c**) T-705; (**d**) T-1106; (**e**) favipiravir form I; (**f**) favipiravir form II; (**g**) inosine; and (**h**) acadesine.

**Figure 12:**
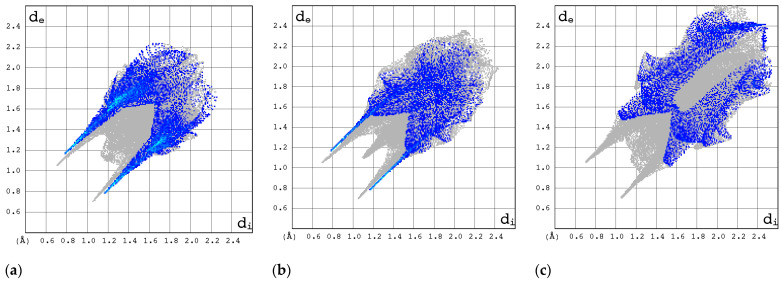

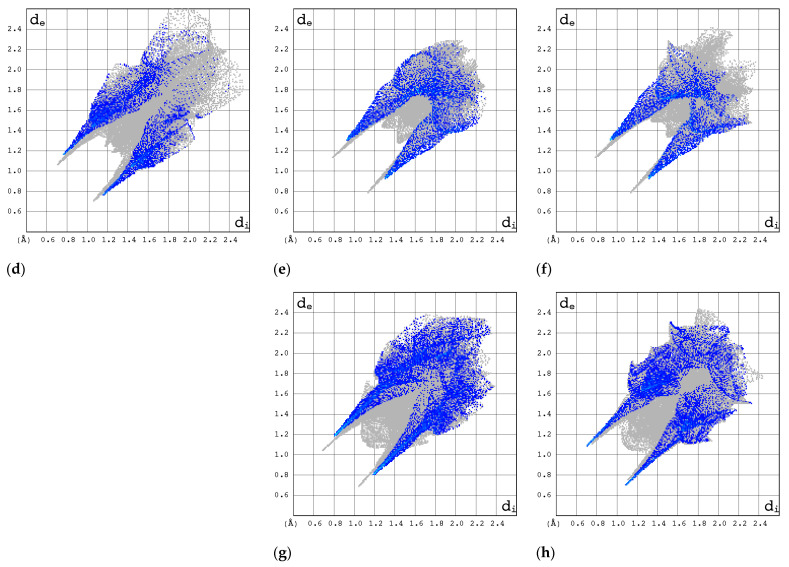
The N···H/H···N contacts visualized in the 2D FP (d_i_/d_e_) plot (the chromaticity of a given hue is expressed on a scale of blue to red, with higher values indicating greater intensity; the boundaries of the full imprint for the entire molecule are shown in gray): (**a**) RBV form I; (**b**) RBV form II; (**c**) T-705; (**d**) T-1106; (**e**) favipiravir form I; (**f**) favipiravir form II; (**g**) inosine; and (**h**) acadesine.

**Figure 13:**
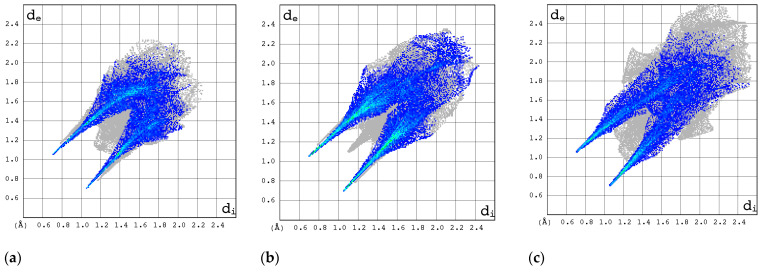

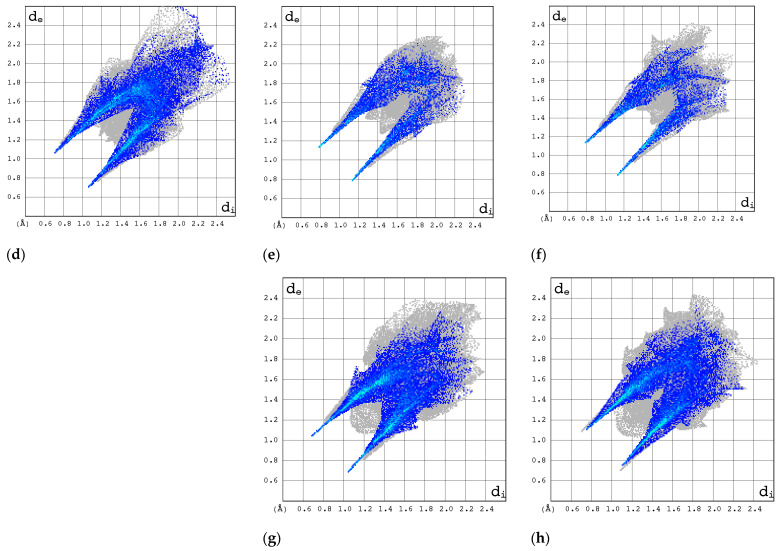
The O···H/H···O contacts visualized in the 2D FP (d_i_/d_e_) plot (the chromaticity of a given hue is expressed on a scale of blue to red, with higher values indicating greater intensity; the boundaries of the full imprint for the entire molecule are shown in gray): (**a**) RBV form I; (**b**) RBV form II; (**c**) T-705; (**d**) T-1106; (**e**) favipiravir form I; (**f**) favipiravir form II; (**g**) inosine; and (**h**) acadesine.

The 2D FP plots, restricted to the most interesting contacts, namely O···H/H···O, C···H/H···C, N···H/H···N, and H···H, as well as F···H/H···F (with the latter only applicable in instances where it is present), are shown in Figure 10, Figure 11, Figure 12 and Figure 13.

In RBV form II, the homonuclear C-H···H-C interactions serve to maintain the specific conformation of the glycone within the crystalline structure. This dispersive interaction, involving the protons at C(5) of aglycone and C(2′) and C(3′) of glycone, exists only within this structure. Indeed, the 2D FP has a particular characteristic—a sharp and narrow spike in the center of the graph in the range of 2.3–3.6 Å.

The C···H/H···C contacts (Figure 11) are weak in all structures. The point cloud is characterized by a high degree of spatial dispersion, a low density of points, and a relatively small overall size.

The N···H/H···N interactions are most pronounced in forms I and II of RBV and T-1106 (Figure 12). The presence of fluorine in the T-705 and favipiravir structures has a deleterious effect on N···H/H···N interactions. In the majority of instances, the 2D FP plot exhibits two distinct and narrow spikes, with the exception of T-705. In the case of T-705, the distance between the contacting atoms of N and H is considerable; thus, their interaction is relatively weak.

The O···H/H···O interactions are most pronounced in form II of RBV, T-1106, and T-705 (Figure 13). The presence of fluorine in the two latter structures has only a subtle effect on their strength. The O···H/H···O interactions represent the most stable and repetitive motif within this series of compounds.

**Figure 14:**
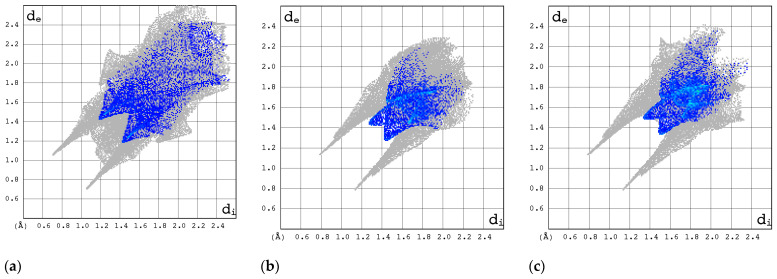
The F···H/H···F contacts visualized in the 2D FP (d_i/_d_e_) plot (the chromaticity of a given hue is expressed on a scale of blue to red, with higher values indicating greater intensity; the boundaries of the full imprint for the entire molecule are shown in gray): (**a**) T-705; (**b**) favipiravir form I; and (**c**) favipiravir form II.

The strongest evidence of the F···H/H···F interactions is observed in form II of favipiravir (Figure 14). In T-1106, these interactions were found to be the least significant. The ribofuranosyl substitution alters the efficiency of the F···H/H···F interactions.

In general, the 2D FPs are comprised of three primary contact types, designated as H···H, O···H/H···O, and N···H/H···N, with different weights across all studied structures. The most significant contribution to the 3D HS of form I of RBV (38.5%) comes from weak H···H interactions, as evidenced by the cloud of scattered points with a pronounced spike at d_e_ + d_i_~2.1 Å (Figure 10). These interactions in form II manifest as a narrow and sharp spike at d_e_ + d_i_~2.1 but are less well represented on the surface (only 31.7% of 3D HS). In form II, the highest contribution to the 3D HS (of 43.2%), which comes from O···H/H···O, is depicted by the most internal and sharp wings at d_e_ + d_i_~2.05. The distinctions between the 2D FPs for the RBV forms are readily discernible and manifest in the configuration and density of point clouds. It is notable that in the case of favipiravir, these discrepancies between the forms are negligible. Concerning the remaining compounds, the O···H/H···O contribution varies from 18.2 to 35.7%, but the distribution of point clouds is less concentrated. The N···H/H···N contacts, which are represented by the most external narrow and sharp wings at d_e_ + d_i_~2.4 Å, account for a mere 8.7–19.8% of the total 3D HS. The contacts encompass smaller regions, which suggests a potential reduction in the efficacy of these interactions. The C···H/H···C contacts are of minor importance across all studied structures. The most external wings at d_e_ + d_i_~3.0 Å serve to illustrate their presence. The proportion of the total 3D HS that they cover ranges from 4.9 to 9.5%. It can be reasonably assumed that the impact of other contributions is insignificant. The presence of the carboxamide has a discernible impact on the total contribution from O···H/H···O and N···H/H···N contacts, whereas ribofuranosyl has the opposite effect. Both ribofuranosyl and carboxamide reduce the contribution from C···H/H···C contacts. The ribofuranosyl moiety exerts a reducing influence on F···H/H···F contacts, and an increasing influence on O···H/H···O contacts.

**Figure 15:**
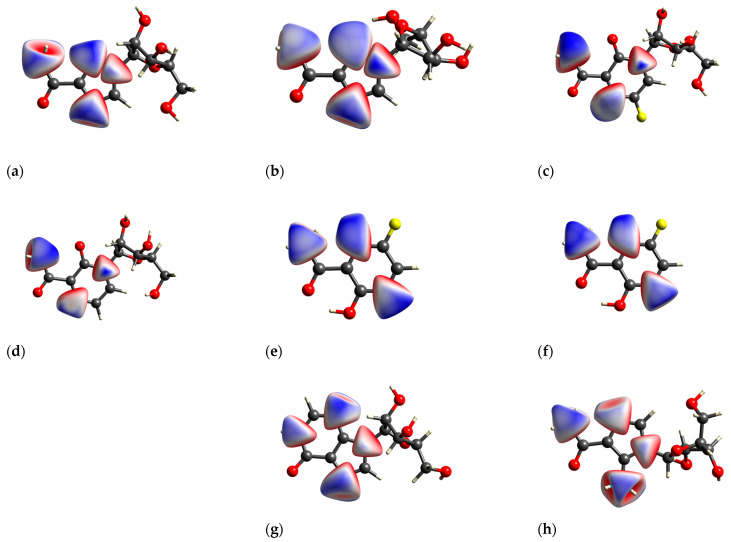
The local 3D HS on nitrogen atoms: (**a**) RBV form I, (**b**) RBV form II, (**c**) T-705, (**d**) T-1106, (**e**) favipiravir form I, (**f**) favipiravir form II, (**g**) inosine, and (**h**) acadesine. A red-white-blue scheme (red: negative, white: zero, blue: positive) was used.

The most intriguing contributions to the 3D HS originate from nitrogen atoms (Figure 15), which is highly interesting from the perspective of experimental data analysis.

The local 3D HS of the nitrogen atoms provides insight into the manner in which they interact with neighboring atoms within the molecule or crystal.

The local 3D HS in the immediate vicinity of >NRy is characterized by significant compression, a pronounced degree of asphericity, and a comparatively low degree of globularity. This outcome directly results from the substitution of ribofuranosyl at N(1). The remaining 3D HSs have relatively similar shapes.

**Figure 16:**
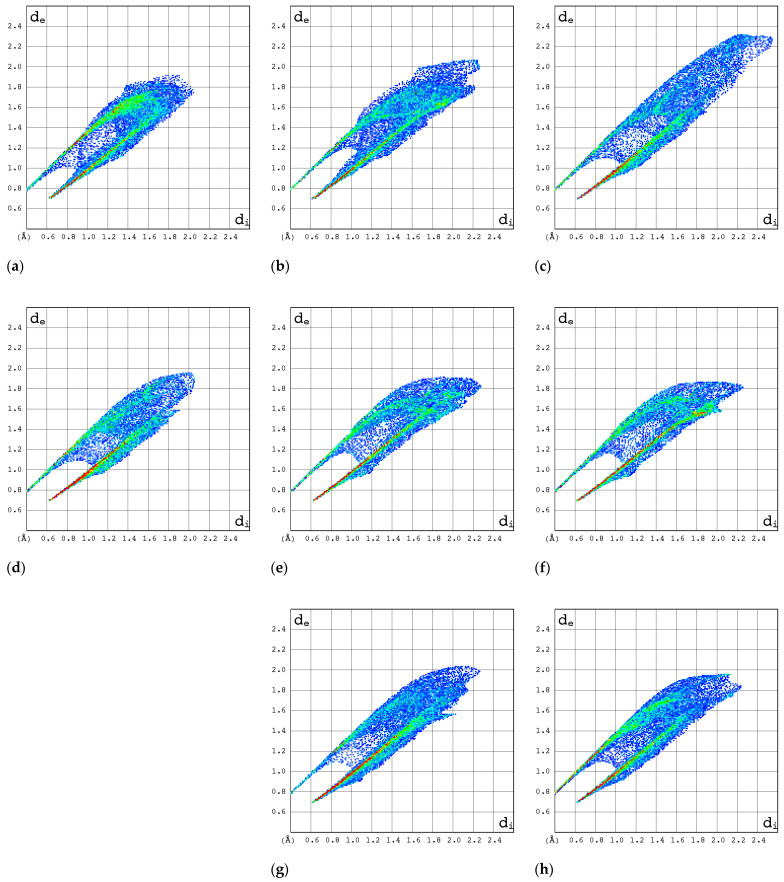
The local 2D molecular fingerprints at all nitrogen atoms in molecule: (**a**) RBV form I, (**b**) RBV form II, (**c**) T-705, (**d**) T-1106, (**e**) favipiravir form I, (**f**) favipiravir form II, (**g**) inosine, and (**h**) acadesine. The chromaticity of a given hue is expressed on a scale of blue to red, with higher values indicating greater intensity.

A detailed examination of the local 2D FPs derived from 3D HSs in the vicinity of all nitrogen atoms in the molecules (Figure 16) provides insights into their involvement in bindings. The differences between 2D FPs are relatively small and cover primarily the region of high d_norm_ and top part of the wing. As demonstrated in the accompanying diagrams, the top wing is indicative of N-H bonds, while the bottom wing is indicative of N-C bonds.

Surprisingly, the 2D FP diagram for RBV demonstrates a higher degree of similarity to T-1106 than to T-705. The analysis of the distances between the percentage contributions to the local 3D Hirshfeld surface at nitrogen sites measured by root mean square deviation, RMSD_N (Table 9), confirms the above observation.

The percentage contributions to the local 3D Hirshfeld surface, RMSD_N, in RBV bear the greatest similarity to those in T-1106, T-705, and favipiravir (form I and form II).

**Figure 17:**
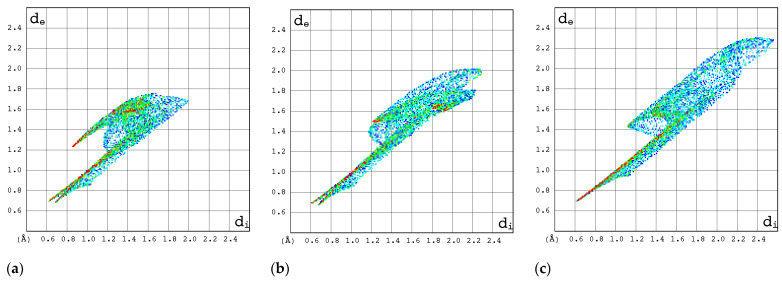

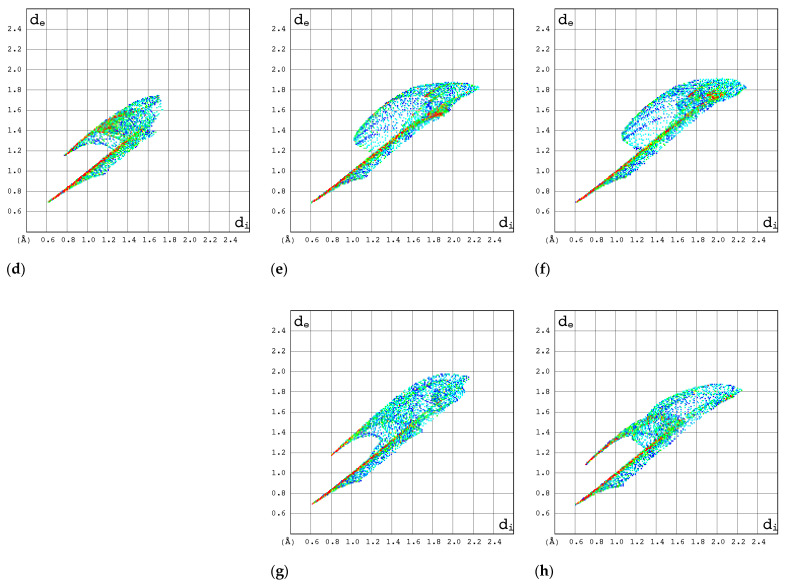
The local 2D molecular fingerprints at –N=: (**a**) RBV form I, (**b**) RBV form II, (**c**) T-705, (**d**) T-1106, (**e**) favipiravir form I, (**f**) favipiravir form II, (**g**) inosine, and (**h**) acadesine. The chromaticity of a given hue is expressed on a scale of blue to red, with higher values indicating greater intensity.

The local 2D FPs in the vicinity of the key –N= nitrogen atom (Figure 17) differ significantly, mainly in the shape of the upper wing. This part of the wing describes the interaction between N and H in the hydrogen bond (inter- or intramolecular).

In the RBV and favipiravir polymorphs, intramolecular hydrogen bonds of the NH···N type are formed, whereas in the remaining compounds, they are replaced by NH···O hydrogen bonds. The intermolecular NH···O bond in form I of RBV (absent in form II) and in T-1106 (absent in T-705) exerts a considerable influence on the shape of the upper part of the 2D FP wing, which assumes a peak-like form characterized by a thin and narrow profile. On the other hand, two narrow but overlapping peaks in the lower part of the wing, visible only in the 2D FP for RBV, are the result of the presence of chemically non-equivalent N-C and N=N bonds, in which –N= participates. It should be noted that this particular feature is also present in other compounds, namely 1*H*-1,2,4-triazole and 1*H*-1,2,4-triazole-3-carboxamide, which possess an N-N moiety. In the NQR parameters, this effect is manifested by a high asymmetry parameter. The two nitrogen–hydrogen bonds of the amine moiety contribute to the 3D HS to an equal extent.

Given that the T-705 and T-1106 conformations of the carboxamide with respect to the pyridazine ring are reversed by 180 degrees, the intramolecular bond is of the NH···O type, which is indicated on the local 3D HS of the oxygen atom (=O). In each of the compounds studied, the quasi-ring is formed as a result of intramolecular NH···N or NH···O bonding, respectively, while in favipiravir, the intramolecular bond is OH···O. Upon ribofuranosyl substitution, the conformation of the carboxyl group is flipped by 180 degrees, and in solid state, this effect seems independent of the type of aglycone. The conformations of the whole RBV molecules in forms I and II are close to T-705 and T-1106, respectively. It should be added that fluorine and ribofuranosyl conformational changes significantly modify the local 3D HS on the N(2) and N(1) nitrogens, respectively.

#### 2.3.4. Intermolecular Interactions in the Solid State

The calculations of intermolecular interaction energies with a graphical representation of their magnitude in the form of energy framework help to understand the topology of the interactions between the constituents of a crystal. It is well known that stronger interactions, such as hydrogen bonding, often influence weak intermolecular interactions in crystals. A detailed analysis of the 3D topologies for the dominant intermolecular interactions in the crystal structures can reveal the significant contributions and their interrelationships. In order to rationalize the mechanical behavior of RBV in the context of polymorphism and explain the similarity of form II of RBV to T-705, this approach seems promising.

An analysis of the total lattice energy and its terms (electrostatic, polarization, dispersion, and repulsion) derived from DFT calculations for a cluster of 10 Å in diameter is presented in Table 10.

**Figure 18:**
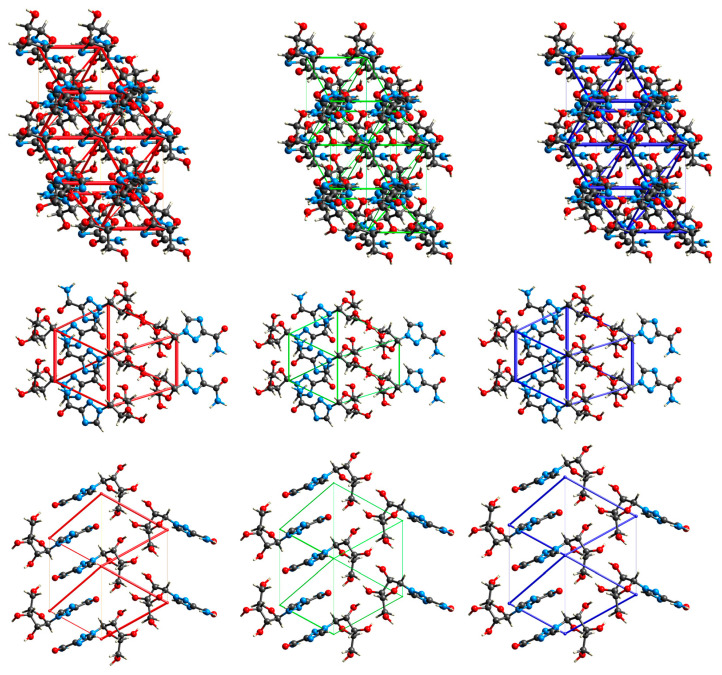
Energy framework diagrams for electrostatic (**left column**), dispersion (**middle column**), and total energy (**right column**) visualized in the RBV form II structure by red, green, and blue “sticks”, respectively (view along the a-axis—(**top**), along the b-axis—(**middle**), and along the c-axis—(**bottom**)). All diagrams use the same energy cylinder scale and the energy threshold.

**Figure 19:**
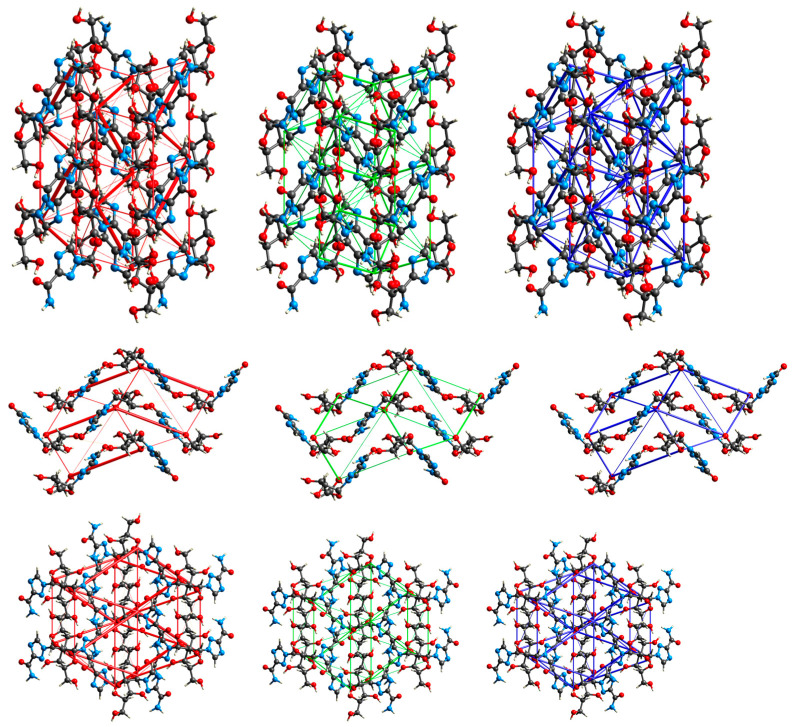
Energy framework diagrams for electrostatic (**left column**), dispersion (**middle column**), and total energy (**right column**) visualized in the RBV form I structure by red, green, and blue “sticks”, respectively (view along the a-axis—(**top**), along the b-axis—(**middle**), and along the c-axis—(**bottom**)). All diagrams use the same energy cylinder scale and the energy threshold.

The highest degree of stability is observed for RBV II, followed by RBV I, inosine (β-form), acadesine, T-705, T1106, 1*H*-1,2,4-triazole-3-carboxamide, favipiravir form I, favipiravir form II, and 1*H*-1,2,4-triazole. It has been established that repulsion and electrostatic interactions are major contributors in both polymorphic forms of RBV. In form I, total lattice energy (−250.79 kJ/mol) is dominated by the electrostatic term (−205.58 kJ/mol, respectively), which is stronger than the dispersion or polarization terms (−138.88 and −46.75 kJ/mol, respectively). Similarly, in form II, total lattice energy (−250.79 kJ/mol) is dominated by the electrostatic term (253.46 kJ/mol), which is stronger than the dispersion or polarization terms (−125.55 and −50.71 kJ/mol, respectively). It is worth noting that in the more stable form II, dispersion is weaker than in form I, whereas repulsive, electrostatic, and polarization terms are stronger (Figure 18 and Figure 19). Dispersive forces are relatively weak; the rings do not form molecular columns, which could serve as the backbone pillars in these structures.

**Figure 20:**
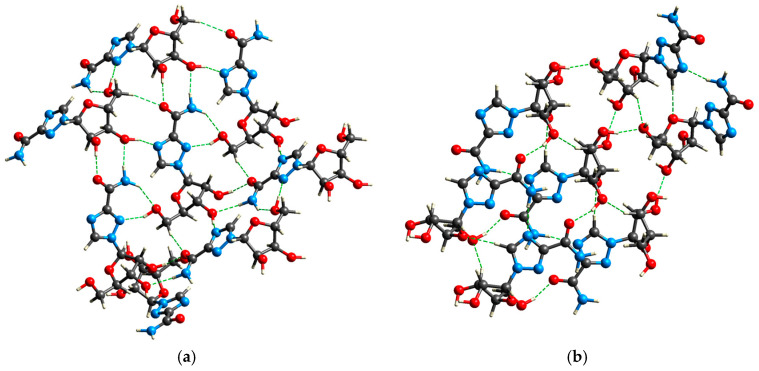
The binding pattern in (**a**) form I and (**b**) form II of RBV in the solid state.

The structure-forming interactions are hydrogen bonds and numerous short contacts between atoms in the interlayers of molecular sheets. The crystalline packing in RBV is determined by mainly electrostatically strong O–H⋯O, N–H⋯O, N−H···N, and C–H⋯O hydrogen bonds (Table 11, Figure 20).

In contrast to form I, the enhanced stability of form II is not attributable to the stronger cohesive energy, but to the presence of more robust hydrogen bonds, especially within the glycone moiety. Form II of RBV is more stable in the solid phase than form I (56.98 kJ/mol).

Moreover, the form II conformation is analogous to the high anti-conformations that have been identified in several effective chemotherapeutic agents, including ortho-azanucleosides (6-azacytidine, 6-azauridine, 8-azacytidine, 8-azatubercidin, and formycin). A salient question pertains to the preferred RBV conformation in the protein–ligand complex and the underlying factors that precipitate this particular preference.

### 2.4. Binding Motifs in the Protein–Ligand Complex

The SARS-CoV-2 genomic arrangement is composed of four structural proteins including nucleocapsid protein (N), spike protein(S), envelope protein (E), and membrane protein (M); 16 non-structural (Nsps); and 9 accessory proteins (ORFs) [64,65,66]. Immediately after infection, the viral RNA is translated to produce a polyprotein, which is then cleaved to produce Nsps involved in genomic/subgenomic RNA transcription. The replication and mRNA transcription complex consist of three Nsps: Nsp12 (RNA-dependent RNA polymerase, RdRp), which is a core and exhibits significant catalytic activity, and two cofactors, Nsp7 and Nsp8. The triad Nsp12-Nsp7-Nsp8 is called the minimal core component, which further binds several additional nonstructural proteins (Nsp9, Nsp10, Nsp13, Nsp14, and Nsp16) [64,65,66]. The catalytic core of the RdRp is localized in the palm subdomain of SARS-CoV-2 RdRp (residues 585–625 and 680–807). The active site of the RdRp polymerase is formed by two catalytic motifs: A (the residues from 611 to 626) and C (residues from 753 to 767). The RdRp inhibitors are known to be involved in binding to the following residues: Asp760, Asp761, Gly616, Trp617, Asp618, Tyr619, Pro620, Lys621, Cys622, Leu758, Ser759, Ala762, Ala797, Lys798, Cys799, Trp800, His810, Glu811, Phe812, Cys813, Ser814, and Gln815. However, three residues play a key role in the inhibition of RdRp, Asp618, Asp760, and Asp761, supported by Asp623, which is responsible for the formation of the hydrogen bond with the 2′-OH group of the nucleoside triphosphate (the sugar selection). The Ser759 residue is involved in the positioning of the priming nucleotide, while Lys798 stabilizes the RdRp core. The nucleotide triphosphate substrates enter the main enzyme channel formed by Lys545, Arg553, and Arg555 residues and form a complex. The RNA template enters the active site composed of motifs A and C.

In our previous research, the binding mode of favipiravir and its halogenated analogues was analyzed in detail [23,24].

For the comparative analysis of bindings, the EM (electron microscopy) structures of SARS-CoV-2 RdRp (Nsp7-Nsp8-Nsp12) in complex with two templates, primer dsRNA and Ribavirin-TP (code 7DFH, [67]), and primer dsRNA and Favipiravir-RTP (PDB code: 7DFG [68]), were retrieved from the Protein Data Bank (PDB). The geometry of the Nsp12 chain in both structures reveals a high degree of resemblance: identity 99.89%, similarity 99.89%, and 0% gaps. (The only difference is the replacement of the Asp (in 7DFH) with Thr (in 7DFG).) A pocket search and 3D pharmacophore analysis carried out using CavityPlus [69] shows strong druggability of cavities in both structures. The maximum and average predicted binding affinity, pK_d_, of the empty cavity are higher for 7DFH than 7DFG (7.91 vs. 7.23 and 6.77 vs. 6.72, respectively). As both are measured for the same cavity and the same protein, the differences can be considered as a measure of statistical spread.

#### 2.4.1. The Role of Conformational Selection in Ligand Binding

**Figure 21:**
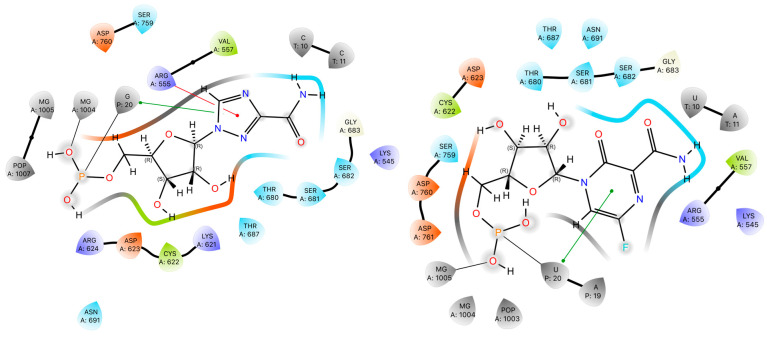
The key protein–ligand interactions in 7DFH (**left**) and 7DFG (**right**).

The conformations of the amide groups in Favipiravir-RTP (FVP-RMP) and Ribavirin-phosphate (RBV-MP) are stabilized by the intramolecular N–H⋯O and N–H⋯N hydrogen bonds of 2.861 and 2.806 Å, respectively. The closing of six and five-member rings, respectively, results in a structure that is reminiscent of guanine and facilitates π⋯π stacking. Amide moiety in FVP-RMP is involved in hydrogen bonds to uracil and adenine, while RBV-MP binds two cytosines (nucleobases from the RNA template strand; Figure 21). The five-membered, aromatic, π excessive and planar ring of RBV-MP is involved in non-covalent π–π stacking to the guanine ring (stack distance: 4.18 Å), whilst the six-membered, aromatic, and planar FVP-MP ring participates in π–π stacking to the uracil ring (stack distance 3.81 Å). The former is weaker than the latter, despite the self-stacking of pure guanine being more stable than pure uracil, which suggests that the dipole moment of FVP is higher than that of RBV.

The amide group of both ligands is involved in binding to Lys545. Additionally, amide of FVP-RTP binds Ser682, but –N(4)= of RBV-MP binds Arg555. The ribofuranosyl group of FVP-RMP binds Asp691, Asp623, Asp 760, and Mg^2+^, while the analogous group of FVP-RMP is responsible for binding Asp691, Thr680, Asp623, Asp760, and Mg^2+^ (Table 12).

In-depth analysis of the binding modes reveals that FVP-RMP is involved in binding with four Mg^2+^, two Zn^2+^, and 137 residues, while Ribavirin monophosphate binds three Mg^2+^, two Zn^2+^, and 119 residues. In RBV-MP Arg555, Mg^2+^, Lys545, Leu854, Glu857, and Asp623 exhibit enhanced binding strength, while Arg858, Ser861, Ser682, and Lys577 demonstrate diminished binding strength compared to FVP. RBV-MP stabilizes the protein–ligand complex more strongly than FVP-RMP (−241.97 vs. −229.93 kJ/mol i.e., by 12.039 kJ/mol). When the differences between the corresponding bonds of residues and cofactors in both complexes are considered, the difference in binding strength is found to be slightly less than 11.496 kJ/mol. The Manhattan distance between binding modes (all bindings included) is 126.78 kJ/mol, while the Euclidean distance is 300.58 kJ/mol. However, the root mean square deviation of binding modes is only 1.32 kJ/mol. The structural disparities between the ligands are substantial, yet the binding mode exhibits a high degree of similarity. The PRODIGY model, which predicts binding affinity based on regression, suggests that FVP ligand produces a more stable complex than RBV-MP (−26.40 kJ/mol vs. −22.38 kJ/mol, for 7DFG and 7DFH, respectively). However, this rough estimation excludes the electrostatic contribution. The evaluation of the whole complex results in binding affinities of −71.977 kJ/mol and −50.345 kJ/mol, for 7DFH and 7DFG, respectively. Thus, the electrostatic term is a highly important component of the binding. The hydrogen bonds term in FVP exhibits greater strength than that of RBV (−21.75 vs. −19.28 kJ/mol for 7DFG and 7DFH, respectively). The question under consideration is whether SARS-CoV-2 RdRp prefers the same conformation of ribofuranosyl when incorporating RBV or FVP into its structure and to what extent this configuration approaches the solid-state conformation.

When looking along the glycosidic bond, a positive rotation corresponds to a clockwise rotation of the far group. Thus, the rotamers with χ angle between −90° and +90° are called anti, whereas syn describes conformation with the χ angle ranging from +90° to +270. The intermediate conformation, with a dihedral angle ranging from 90° to 130°, is designated as the high-anti conformation. The high-syn conformation corresponds to the intermediate range from 270° to 310°. In the solid state, the predominant configuration is the high-anti conformation (Table 13). In solution, however, the high-syn form of RBV-MP, which is not biologically active, predominates [70]. As demonstrated in Table 13, known protein–ligand complexes with RBV, RBV-MP, and RBV-TP exhibit anti- or high-anti conformations.

However, high-anti is only present in form II and 3FSU (*Murine norovirus*, RdRp with RBV) complex and is scarce overall. The full geometry optimization of the RBV structure does not exert any influence on the conclusions that are derived. Both complexes of RdRp of SARS-CoV-2 with FVP-RMP and RBV as ligands (7DFG and 7DFH, respectively) adopt a conformation of anti, instead of high-anti. This contrasts with the predominant high-anti conformation observed in solid or high-syn conformation that predominates in solution. In addition, it has been observed that the angle between the rings, θ, in the two complexes and 2′-O-methyltransferase of *Denga* virus with RBV-MP (1R6A) is nearly half of that observed in the remaining structures.

It is well known that glycosidic torsion angle χ is correlated with sugar puckering. In all studied structures, the glycone moiety maintains C3′-endo sugars’ conformation (A-helix; Table 13). However, as is known, high-anti is often found with C2′-endo sugars (B-helix), while C3′-endo sugars (A-helix) are associated with more canonical anti (θ values in the range from −150° to −180°). With the exception of RdRp of *Murine norovirus*, the RNA primer demonstrates a preference for the configuration anti. This is consistent with the observation that the high-anti range is often populated in duplex structures and is therefore preferred by DNA. This disparity is attributable to the hydroxyl group at the 2′ position within the RNA glycone, which facilitates interaction with the backbone or other bases. The 2′-OH shifts the conformational equilibrium of the five-membered glycone ring toward the C3′-endo pucker. Nevertheless, no correlation has been identified between conformational characteristics and the binding energy of diverse protein structures.

The root mean square deviation of atomic positions, RMSD, between the RBV ligands in the complex and in the solid state is 8.17 and 7.37 Å for form II and form I, respectively. In contrast, the RMSD between both forms, I and II, is only slightly smaller and equal to 6.99 Å. Upon analysis of the binding mode, it becomes evident that there are certain common features of RBV binding that are consistent with those observed in the solid state and protein–ligand complexes. In the solid phase of RBV, in form I, the amide group participates in strong NH⋯O and OH⋯O hydrogen bonds, while in form II, the amide group forms strong NH⋯N and weaker NH⋯O hydrogen bonds and is involved in weak dispersive NH⋯HC contact. In form II, unlike form I, the glycone moieties bind together by OH⋯O bonds. In contrast, the basic binding motifs in protein–ligand complexes with RBV as ligand are strong NH⋯N and NH⋯O hydrogen bonds, while glycone moieties do not bind to each other. From the point of view of the biological activity, the ability to bind the amide group of aglycone seems much more important than three hydroxyl moieties of glycone, whereas the glycone is required for RBV incorporation into the RNA primer.

#### 2.4.2. The Role of Structural Similarity in the Binding of the Ligands

The six ligands shown in Figure 1 were incorporated into RNA primer, combined with TNA template, and docked into the binding site in RdRp that had been previously prepared by correcting protonation and atomic hybridization. The protein from the 7DFH structure was used for docking, and both A and C chains were considered. The docking results are listed in Table 14.

**Figure 22:**
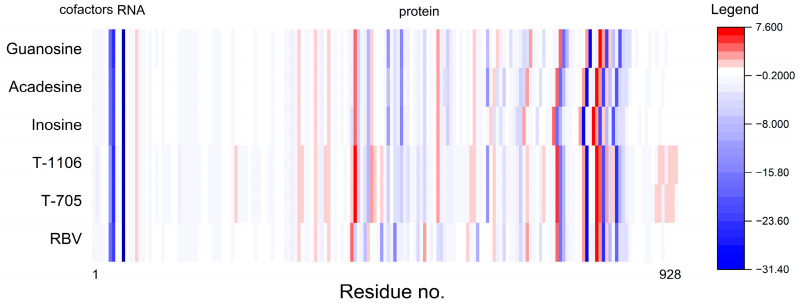
The protein–ligand binding modes visualized using heatmaps (strong stabilizing interactions are shown in blue, while destabilizing complex is shown in red).

The protein–ligand binding modes are visualized in Figure 22. The blue is used to denote strong interactions that stabilize complexes, while red is used to depict weak de-stabilizing interactions. Residues 7–127 belong to the C chain, and those above 127 to the A chain; low numbers 1–6 correspond to the cofactors.

**Figure 23:**
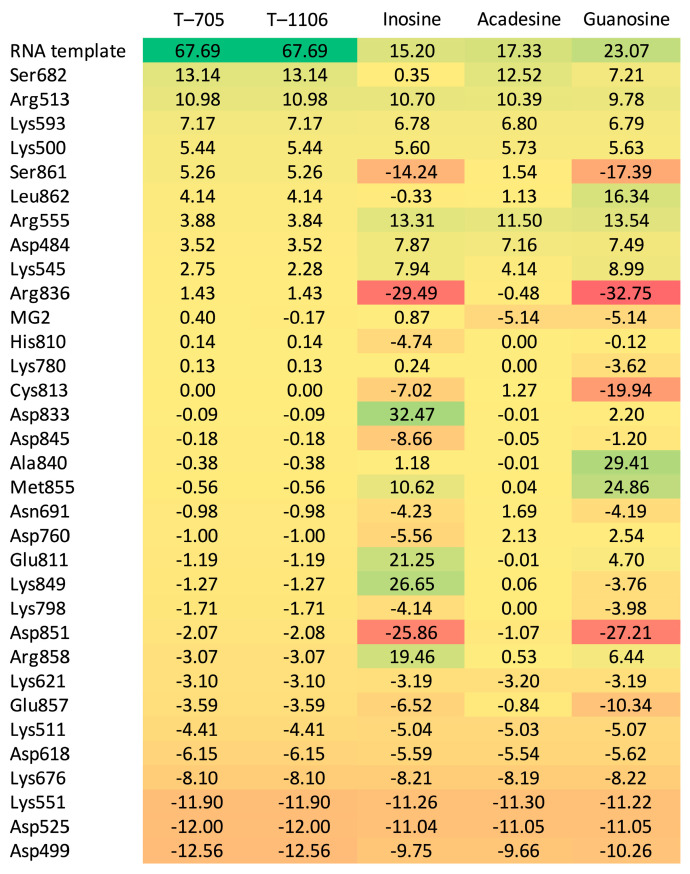
The heatmaps of differences in binding energy (in kJ/mol) between RBV and ligand for selected residues (red represents the negative values, while green indicates positive values); RBV is the reference for five ligands (The green-yellow-red scheme, with dark green indicating stronger binding and dark red indicating weaker binding, was used).

As can be seen from Table 14 and Figure 23, T-705 and T-1106, unlike inosine, acadesine, and guanosine bind the RNA template more strongly than RBV but bind cofactors—magnesium Mg^2+^ ions—slightly more weakly. The binding mode of RBV, T-705, and T-1106 is clearly different from the other three.

The acadesine binding mode bears a striking similarity to the RBV binding mode. The color-coding system utilizes green marking for residues that bind a given ligand more strongly than RBV, and red marking for residues that bind the given ligand less strongly than RBV. Inosine binds residues Asp833, Lys849, Glu811, and Arg859 noticeably more strongly than RBV, whereas residues Arg836, Asp851, and Ser 861 are bound much weaker. The binding mode of guanosine and inosine is similar. Nevertheless, certain residual elements, including Asp833, Glu811, Lys849, Arg85, Ala840, Leu862, and Met855, exhibit discrepancies. The root mean square distance between binding modes (RMSD_BM) and the Manhattan distance for the entire binding modes (Table 15) also lead to the conclusion that the protein residues bind acadesine in a manner most similar to RBV. Moreover, a high resemblance is observed between T-705 and T-1106.

The docked ligands arranged according to their binding affinity, with the most strongly bound ligand occupying the first position, are listed in Table 16. The binding affinity varies from −29.84 to −71.98 kJ/mol. Both T-705 and T-1106 exhibit significantly stronger binding affinities in comparison to the other ligands under consideration.

Table 16 also provides a list of the novel SBAI indices, which are calculated on the assumption of three different similarity factors (Tanimoto similarity, atom pair Tanimoto similarity, and maximum common substructure similarity). The smaller the structural difference between the ligand and reference (RBV), the higher the values; for the ligand identical to the reference, indices are infinitely large. SBAI makes a division of the ligands into two distinct groups: strongly binding (active, SBAI > 0) and weakly binding (non-active, SBAI < 0). The degree of similarity between the ligands measured by different similarity factors varies from 44 to 84%.

**Figure 24:**
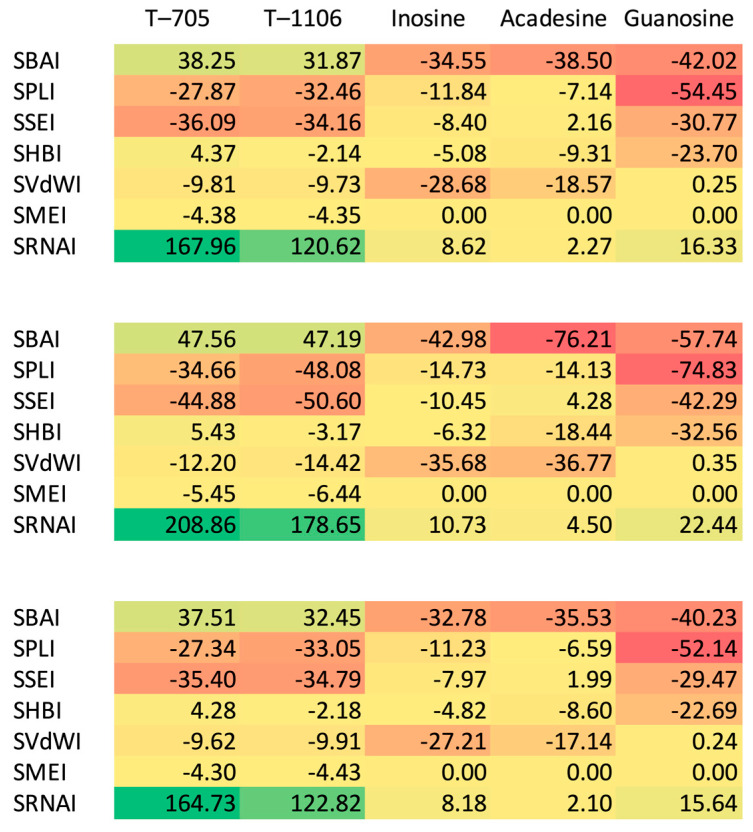
The heatmaps of structure—binding strength indices (structure—binding affinity index, SBAI; structure—protein–ligand index, SPLI; structure—steric effect index, SSEI; structure—hydrogen bond index, SHBI; structure—van der Waals, SVdWI; structure—metallic bond index, SMEI; and structure—template RNA binding index, SRNAI). Red represents the negative values, while green indicates positive values; (**top**): Tanimoto similarity, (**middle**): atom pair Tanimoto similarity, and (**bottom**): maximum common substructure similarity) (The green-yellow-red scheme, with dark green indicating more active and dark red indicating less active, was used).

A rough analysis of the structure—binding strength indices (SBSI), shown in Figure 24, suggests that T-705 represents the best choice.

As demonstrated in Figure 24, there is a clear delineation between the two distinct groups, a phenomenon analogous to that observed in Figure 2. The division into two groups indicated by yellow-green and yellow-red corresponds to their antiviral activity and non-activity, respectively.

### 2.5. The Novel Quadrupolar Indices (QI)

The considerations set out in the preceding paragraphs were utilized to facilitate the analysis of the binding motifs in crystals and protein–ligand complexes. Their quantification is an essential step towards understanding the mechanisms underlaying biological activity. The fundamental question that remains to be resolved is how to precisely numerically evaluate the differences in the binding abilities of atoms in the solid state and in the protein–ligand complex. The most accurate parameters describing the distribution of electron density on an atom are the quadrupole coupling constant and the asymmetry parameter. To assess differences between the solid state and protein–ligand complex, we defined in this paper a set of new parameters, the so-called quadrupolar indices (QI). Four of the indices describe binding motifs with respect to the structure of a single molecule, while two others characterize the difference between the binding mode in the solid state and in the protein–ligand complex. They can be treated as helpful metrics in the evaluation of solid-state effects and complexation effects and for clarifying the differences between the solid state and the complex.

#### 2.5.1. The Quantification of the Solid State and Protein–Ligand Complexation Effects

Four indices describing the shifts in NQR parameters facilitate the evaluation of both solid-state and protein–ligand complexation effects from the ligand perspective. The following definitions are hereby proposed for the indices:

(4) $$\Delta^{s}={\left|\frac{e^{2}qQ}{h}\right|}_{solid\;state}-{\left|\frac{e^{2}qQ}{h}\right|}_{single\;molecule}$$

(5) $$\delta^{s}=\eta_{solid\;state}-\eta_{single\;molecule}$$

(6) $$\Delta^{c}={\left|\frac{e^{2}qQ}{h}\right|}_{protein-ligand\;complex}-{\left|\frac{e^{2}qQ}{h}\right|}_{single\;molecule}$$

(7) $$\delta^{c}=\eta_{protein-ligand\;complex}-\eta_{single\;molecule}$$

The indices for studied compounds are listed in Table 17.

As follows from Table 17, hydrogen bonding significantly changes the NQR parameters at the amide nitrogen, –NH_2_, as well as N(4). Thus, both sites in RBV and FVP play an important role in the solid-state binding and should be involved in hydrogen bonds’ formation. The significant change in the δ^s^ at the -NH_2_ suggests that the hydrogen bonds involving the –NH_2_ protons in form II of RBV have different strengths. The opposite signs of the Δ^s^ and δ^s^ indices at the >N-sugar suggest different conformations of the glycone. Very small values of the indices for N(2) and N(4) in form II of RBV compared to form I suggest that N(2) and N(4) in form II do not participate in strong intermolecular bonds. The indices Δ and δ for forms I and II of FVP are much smaller.

Analogous analysis for the protein–ligand complex (Table 18) leads to the conclusion that the most important role in the binding mode is played by –NH_2_ and –N(2)=.

However, the complexation significantly modifies the symmetry of the density distribution at the amide and >N-sugar nitrogen atoms. Furthermore, the lowest Δ ^c^ and δ^c^ indices at N(4) suggests that while it participates in hydrogen bonding within a solid-state framework, such participation is notably absent in the protein–ligand complex.

#### 2.5.2. The Quantification of the Similarity Between Binding Motifs in the Solid State and Protein–Ligand Complex

To determine the binding similarity between a solid and a protein–ligand complex, dedicated indices can be defined as follows:

(8) $$\Delta^{cs}={\left|\frac{e^{2}qQ}{h}\right|}_{protein-ligand\;complex}-{\left|\frac{e^{2}qQ}{h}\right|}_{solid\;state}$$

(9) $$\delta^{cs}=\eta_{protein-ligand\;complex}-\eta_{solid\;state}$$

The corresponding values calculated are listed in Table 19.

The indices listed in Table 19 suggest that the binding motif in the protein–ligand complex is much closer to that of the solid-state form II than to that of the solid-state form I.

Elevated indices at N(1) of FVP are the consequence of ribose substitution exclusively within the protein–ligand complex. Apart from the effect of ribose substitution, the similarity of binding modes in the solid state and protein–ligand complex is much greater for FVP than for RBV. Relatively low values of the Δ^CS^ indices of the nitrogen of the -NH_2_ group indicate its involvement in slightly different hydrogen bonding in the solid phase than in the protein–ligand complex. Indeed, the –NH_2_ group is involved in two NH···O bonds in form I, and in NH··O and NH···N bonds in form II. The much larger, in absolute value, Δ^CS^ and δ^CS^ indices of N(2) in form I result from its involvement in the N···OH hydrogen bond (only in form I). This small difference in indices at sugar-substituted nitrogen in form II of RBV suggests that electron density is equally stabilized in solid phase and complex. A distinct conformation is responsible for the elevated values of indices of sugar-substituted nitrogen in form I of RBV. Furthermore, the highest indices of N(4) suggest the large difference between the solid state and protein–ligand complex. Indeed, N(4) participates in hydrogen bonds N(4)…HO (form I of RBV) and N(4)…HN (form II of RBV) in the solid state, but such bonds are notably absent in the complex. It is evident that a marginal discrepancy exists in the indices of the corresponding nitrogen atoms for the two polymorphic forms of FVP. However, a significant discrepancy is apparent for both polymorphic forms of RBV, which can be attributed to the effect of different ribose conformations.

### 2.6. The Binding Mode Visualization

QTAIM (quantum theory of atoms in molecules) [28,29] analysis can be used to visualize the binding mode and verify the conclusions drawn based on the new indices. The reduced density gradient (RDG), s(r), a scalar field of the electron density, is defined as follows:

(10) $$s\left(r\right)=\frac{\left|\nabla \rho (r)\right|}{2{\left(3\pi^{2}\right)}^{\frac{1}{3}}{\rho (r)}^{4/3}}$$

**Figure 25:**
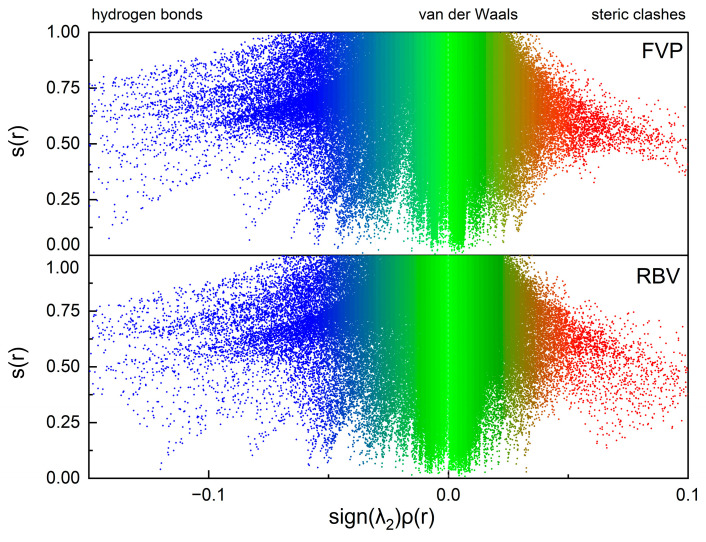
The RDG vs. sign(λ_2_)ρ_BCP_ FVP (**top**) and RBV (**bottom**). The hydrogen bonds, van der Waals, and repulsive non-bonding interactions are depicted in blue, green, and red, respectively.

This is a simple function of the electron density ρ(r) and its gradient ∇ρ(r), describing the deviation from a uniform electron distribution [30]. The sign[λ_2_(r)]ρ(r) variable is defined as a product of the second eigenvalue of the principal component of a Hessian matrix, and ρ(r) is the electron density at the bond critical point. The RDS property is instrumental in facilitating the discernment of weak or delocalized interactions, which can be associated with the regions of small, reduced density gradient at low electron density (Figure 25).

The numerous attractive and repulsive weak non-bonding interactions are indicated by the spikes at positive and negative sign[λ_2_(r)]ρ(r) values. The region demarcated in green, with sign[λ_2_(r)]ρ(r) values that approximate zero, is indicative of a high prevalence of van der Waals interactions. These interactions are found to be slightly stronger in RBV than in FVP. For both compounds, attractive interactions predominate over repulsive ones. Both FVP and RBV exhibit a high number of strong hydrogen bonds; however, for RBV, repulsion (steric clashes) is found to be stronger.

**Figure 26:**
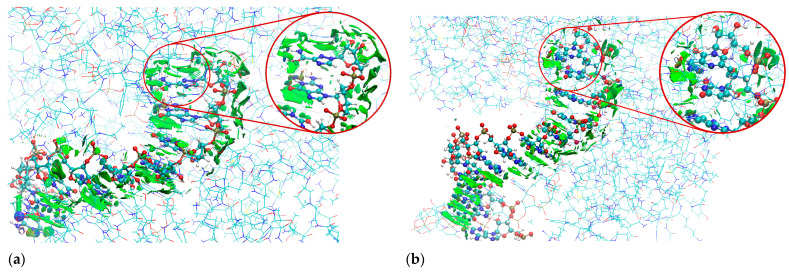
The isosurface of RDG (isovalue 0.5 a.u.) with sign(λ_2_)ρ_BCP_ mapped over the surface, revealing binding modes in the protein–ligand complex with (**a**) RBV and (**b**) FVP-R as ligand. The color scale is from −0.07 (red) to 0.07 a.u. (violet). As illustrated by the green regions, van der Waals interactions are represented, while the cyan disks denote the presence of hydrogen bonds linking the RNA primer with incorporated ligand and RNA template.

The sign[λ_2_(r)]ρ(r) surface mapped to the reduced density gradient, RDG(r), isosurface in the red–green–blue scheme for the protein–ligand complex is shown in Figure 26, visualizing the protein–ligand binding mode.

It reveals the nature of the non-covalent interactions between the proteins and ligands. Weak van der Waals-type interactions dominate, which is indicated by the green color of the surface. The large and nearly flat green region above the ligands’ aglycone ring proves π⋯π-stacking between the ligand and nucleobases. The N–H⋯O and N–H⋯N hydrogen bonds are depicted by a small light cyan disc-shaped region near the O and N of the amide group. The ribbon-shaped region below them suggests weaker binding between RVD and cytosine than that between guanine and cytosine. Furthermore, the RDG(r) surface reveals that N(4) does not participate directly in the binding. The key role in the binding of the ligand (incorporated to RNA primer) in the complex is played by the amide group and glycone, which explains why replacing pyridazine with triazole does not significantly affect the binding affinity.

### 2.7. Molecular Dynamics Simulations

#### 2.7.1. Protein Flexibility

**Figure 27:**
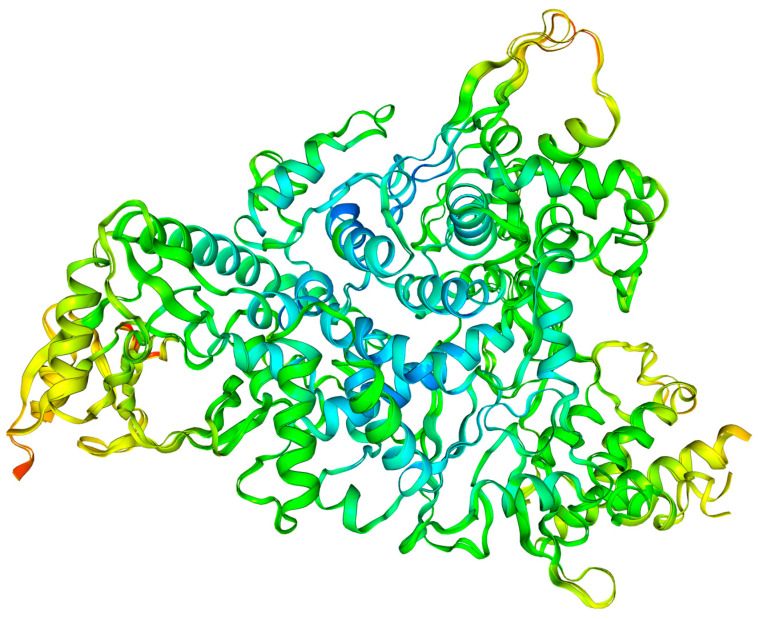
The superimposed structures 7DFH vs. 7DFG. The normalized B’-factors (Debye–Waller factor/atomic displacement parameter) were used as a measure. The more rigid fragments are indicated in red, while the less rigid ones are indicated in blue (scale: red +4, green: 0, blue −2).

The limited flexibility of residues is the weak point of any classical analysis. The fundamental question that must be addressed is whether active sites demonstrate flexibility. The combined Debye–Waller B’-factor [78,79] analysis and molecular dynamics simulations (MDS) provide helpful tools for a such purpose. It is notable that, usually, only the backbone residues exhibit flexibility, whereas the active site residues remain rigid. Nevertheless, there are exceptions to this general rule. The overlay of structures 7DFH and 7DFG (Figure 27) is conducive to the identification of regions that exhibit an increase in rigidity and flexibility. It can be seen that 7DFG, determined with a slightly higher accuracy than 7DFH (2.70 vs. 2.97 Å), displays greater flexibility, particularly in the region of the residues important for binding. In the catalytic loop, the stiffness increases significantly due to the binding between the glycone of the ligand and residues of the target.

**Figure 28:**
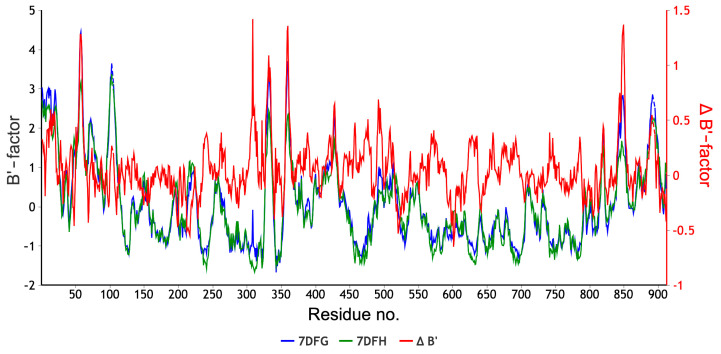
The normalized B’-factors (Debye–Waller factor/atomic displacement parameter) and delta B’. The B’-factor for 7DFH (Ribavirin-TP) and 7DFG (Favipiravir-RMP) are depicted in dark green and blue, and their difference, ΔB’, is shown in red. The key region for binding is depicted with a blue frame.

**Figure 29:**
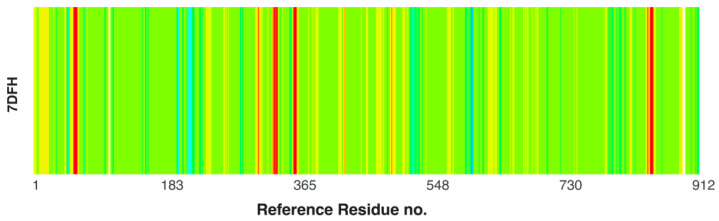
Heatmap visualizing B’-factor alignment. The color scale shows mean absolute deviation (MAD). The red bands indicate rigidification in 7DFH with respect to the reference structure 7DFG.

The diagrams shown in Figure 28 and Figure 29 facilitate the identification of these specific protein regions. A negative B′ value is indicative of higher rigidity than the average of the structure. Conversely, a positive B′ value is indicative of lower rigidity.

The variation in the normalized B’-factor values, denoted by ∆B’, for these two structures falls within the range from −0.5 to 1.5. The comparison of the rigidity of 7DFH and 7DFG shown in Figure 28 and Figure 29 reveals that the replacement of FVP with RBV results in an increase in the stiffness in the regions of Glu61, Phe313, Asp336, Val338, Ser364, Gly431, Asm496, Val848, and Leu854. All of them are distant from the active site. It appears that the binding mode remains unaffected by the ligand flexibility.

#### 2.7.2. Molecular Dynamics Simulation

Molecular dynamics simulation (MSD) has the capacity to provide supplemental flexibility data. This method is vital for analyzing crystallographic structures and providing stability data on complexes from the docking process. The utilization of coarse-grained techniques in MD simulations serves to elucidate the question of active site flexibility.

**Figure 30:**
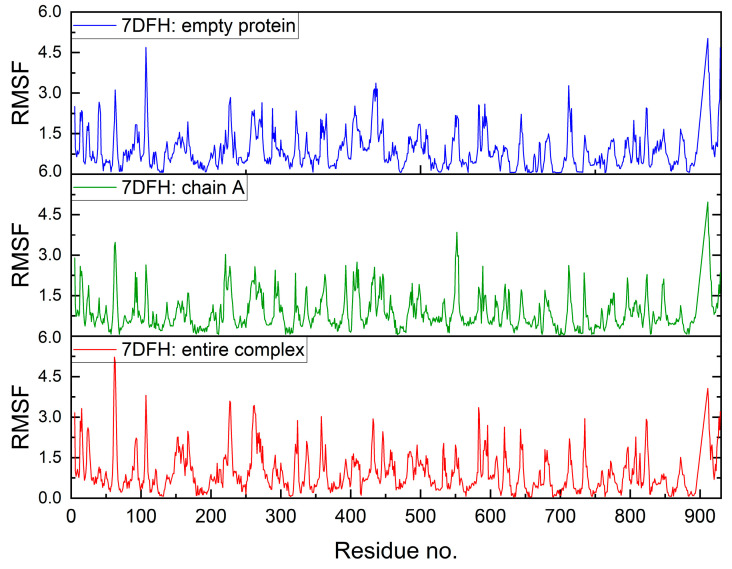

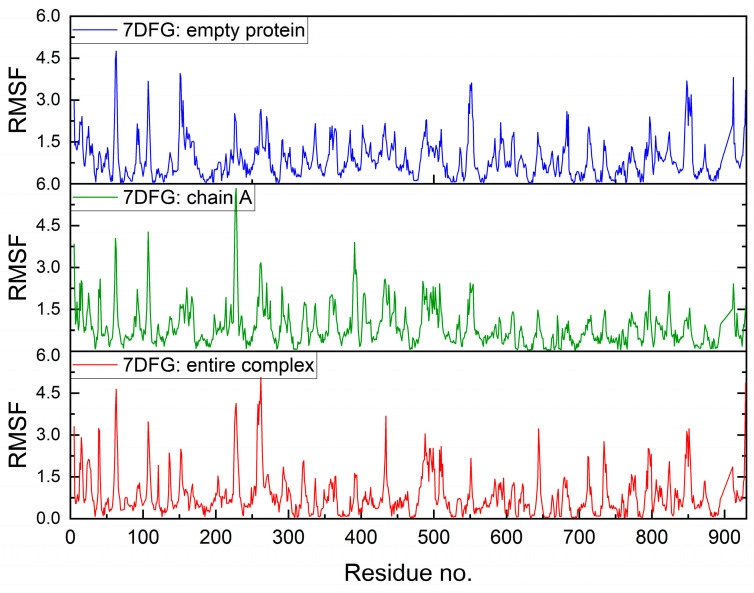
The root mean square fluctuations (RMSFs) for the empty protein, protein chain A-ligand complex, and entire protein–ligand complex as a function of residue number: ribavirin (**top**) and favipiravir (**bottom**).

The assumption of a ligand-depleted protein (a protein with an empty cavity) and the entire protein–ligand complex facilitates an investigation into the impact of the ligand. The root means square fluctuation (RMSF) of a structure, i.e., the root mean square deviation of atomic positions from their mean positions over time (RMSD), is shown in Figure 30. This graph reveals the dynamics of the ligand-depleted protein, the protein chain A-ligand complex, and the complex in its entirety.

**Figure 31:**
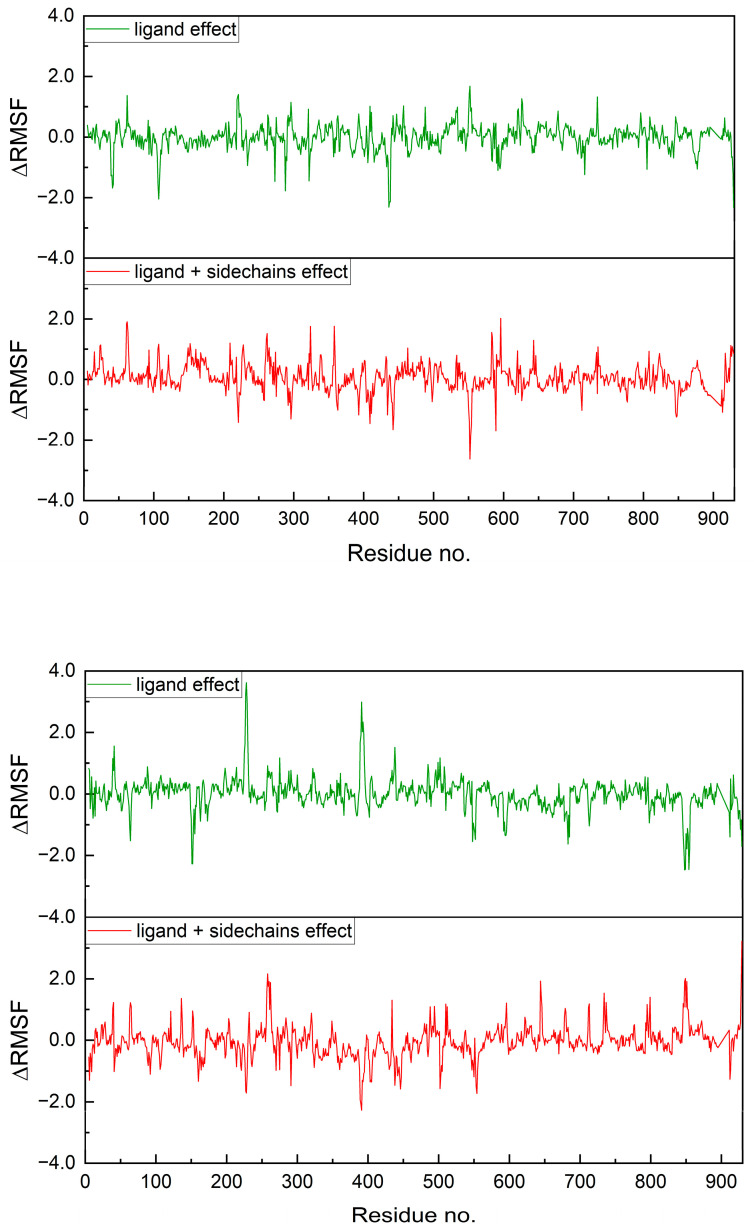
The difference in the root mean square fluctuation (ΔRMSF) between protein–ligand complex and empty protein as a function of residue number: ribavirin (**top**) and favipiravir (**bottom**).

The binding site in RdRp is relatively stable even in the absence of a ligand (empty pocket case). In general, the RMSF value does not exceed 6 Å. The increased flexibility is only evident in the regions of the selected residues (Figure 30). Nonetheless, the aforementioned plots are not conducive to analysis. Therefore, difference plots, which show the differences in RMSF, were generated (Figure 31). They reveal the ligand effect and ligand and sidechains effect.

The presence of ligands exerts a stiffening effect on the entire complex. It is noteworthy that FVP demonstrates a significantly lower propensity to stiffen the overall complex compared to RBV (Figure 31). The maximum difference in ΔRMFs is almost twofold. The flexibility of the remaining residues in other protein chains is not a significant factor in the binding process. However, the remaining chains in the protein serve to compensate for the ligand effect, a phenomenon that is clearly visible in the diagrams as peaks with opposing sign amplitudes. The radius of gyration changed relatively little, by no more than 4%.

**Figure 32:**
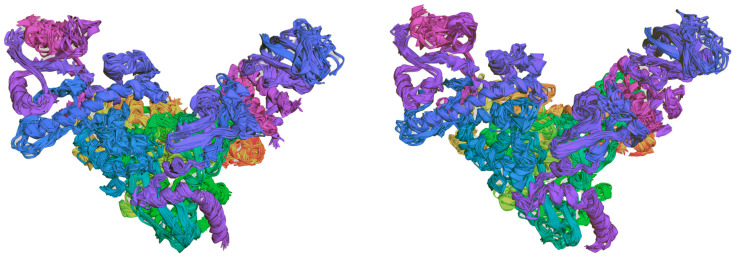
The multi-model visualization (several models superimposed) of the fluctuations of the RdRp complex with ribavirin (**left**) and favipiravir-RMP (**right**).

In Figure 32, the multi-model visualization of the fluctuations (several main models superimposed) is shown.

As illustrated in Figure 30, Figure 31 and Figure 32, the 7DFH and 7DFG complexes exhibit a high degree of stiffness and highly resemble each other.

Consequently, the findings of this study demonstrate that the dynamics exert a negligible effect on the binding mode.

## 3. Material

The powdered sample of RBV (CAS: 36791-04-5) was purchased from Merck Sharp & Dohme, Ljubljana, Slovenia. Its purity was confirmed to be above 98%. The PXRD study confirmed the presence of form II of RBV in the tested sample. An NQR experimental study was conducted, with the RBV sample undergoing no prior recrystallisation or additional purification procedures. The powdered sample was degassed and sealed in a glass ampoule. The experimental conditions were selected with the objective of preventing conversion to form I or hydration.

## 4. Methods

In general, the range of techniques employed is consistent with the methodologies previously implemented and delineated [1,23,26,80]. Nevertheless, it should be noted that some modifications have been made to the instrumental and calculation approaches.

### 4.1. ^14^N Nuclear Quadrupole Resonance (NQR)

The ^14^N NQR spectra were detected at room temperature (295 K) using ^1^H-^14^N cross-relaxation NQR spectroscopy [25,26,42] on a Stelar 0.5T fast field-cycling (FFC) relaxometer.

The FFC technique involves the variation of the primary magnetic field during the pulse sequence. In a standard cross-relaxation (CR) experiment, three successive periods with different static magnetic field strengths are used: a polarization field, a relaxation (mixing) field, and an acquisition field.

By repeating the experiment at different relaxation field strengths B and relaxation times, one obtains the full dispersion of the spin-lattice relaxation time in the mixing field, *T*_1*H*_(*B*). When the proton NMR Larmor frequency *ν*_*H*_ coincides with one of the (Zeeman-shifted) ^14^N NQR frequencies, a reduction of *T*_1_(*B*) (often termed the “quadrupole dip”) is observed [25,26,42].

Because the relaxometer is primarily designed to measure relatively fast relaxation in liquid and soft materials, the cooling of the main coil is not sufficient for very long high-field polarization times. As a result, it was not feasible to acquire a complete T_1H_(B) dispersion curve in ribavirin, which exhibits a very slow relaxation (T_1H_~40 s). Instead, as mentioned in Section 2.1.2, both quadrupole dip and quadrupole peak curves (Figure 3) were determined by simply measuring the amplitude of the signal following a single, typically 10 s period in the relaxation (mixing) field.

Finally, by analyzing these quadrupole dips at Zeeman-shifted NQR frequencies, the pure ^14^N NQR frequencies were extracted using the methods outlined in references [25,26,42].

### 4.2. Density Functional Theory

The cluster calculations based on the NQR-dedicated approach [47] were performed using Gaussian 16, rev. C01 [81], at the M062X/6-31+G(d,p) [82] level of theory. The IR frequency criterion was used to validate the geometry.

All solid-state quantum chemical calculations were carried out within the CASTEP [83] code. The nonlocal generalized gradient approximation (GGA) functionals depending on both ρ and dρ/dr, namely RPBE (revised Perdew, Burke, and Ernzerhof) [84,85]. The Tkatchenko and Scheffler approach [86], which scales the dispersion coefficients on the basis of a Hirshfeld partitioning of the DFT electron density, was used. The use of the gauge, including projector-augmented waves (GI)PAW [43] exploiting the full translational symmetry of a crystal and on-the-fly generation ultrasoft (OTFG) potentials, provided an excellent balance of speed and accuracy. The sampling of the Brillouin zone was carried out with the Monkhorst and Pack [87] scheme (reciprocal space-based technique) using 1 × 4 × 3 k-point separations. Because the components of the electric field gradient (EFG) tensor are highly sensitive to the structure quality, especially positions of the hydrogen atoms, their positions in the RBV structure were optimized. Additionally, full optimization of the polymorph structures of RBV was performed to eliminate possible refinement errors in three-dimensional geometry and assess the quality of X-ray data. The convergence tolerances were chosen to be: SCF criterion at 0.05 eV/atom, force at 0.05 eV/Å, stress at 0.1 GPa, and displacement at 0.002 Å. The cutoff of 571.40 eV was chosen. The LBFGS algorithm [88] that accelerates geometry optimization for large systems and ensures fulfillment of the Born criteria was applied. The IR spectrum was calculated, and the Born criterion for the stability of an unstrained crystal was checked.

The electric field gradient (EFG) tensor components, a second-rank symmetrical tensor, $q_{ii}=\frac{\partial^{2}V(r)}{\partial x_{i}^{2}}$ (i = x, y and z; V(r)—external electrostatic potential, satisfying the relationship $\left|q_{xx}\right|\leq \left|q_{yy}\right|\leq \left|q_{zz}\right|$), were calculated at the selected level of theory. The EFG tensor is fully described by the three eigenvalues and the three eigenvectors, which describe the orientation of the principal axes with respect to an arbitrary frame. However, the ^14^N NQR frequencies depend on NQR parameters: the quadrupole coupling constant e^2^qQ/h = e^2^q_Z_Q/h and the asymmetry parameter η, which are a priori necessary for their determination. Both are related to the ^14^N NQR frequencies according to Equations (1) and (2), where a nuclear quadrupole moment for ^14^N equal to 2.044 fm^2^ is assumed [89].

Conversely, the ^14^N NQR resonant frequencies are associated with these parameters through a series of equations:


(11) $$\nu_{+}=\left|\frac{e^{2}Qq}{4h}\right|(3+\eta )$$

(12) $$\nu_{-}=\left|\frac{e^{2}Qq}{4h}\right|(3-\eta )$$

(13) $$\nu_{0}=\nu_{+}-\nu_{-}=\left|\frac{e^{2}Qq}{2h}\right|\eta$$

The EFG tensor values were calculated assuming several structural variants. For the interpretation of NQR spectra, the EFG tensor values were calculated assuming X-ray structures with optimized hydrogen atoms and fully optimized structures (fixed unit cell). To confirm the frequency assignment, calculations were performed assuming six-molecule clusters of crystals. To determine the quadrupolar indices, complementary calculations were performed, assuming single molecules extracted from the crystal and a fragment of the complex containing RBV/FVP as a ligand.

The calculation level M-062X/6-31+G(d,p) was selected exclusively for six-molecule clusters. The remaining calculations were conducted at the GGA/RPBE level of theory. (As stated in the results and discussion, the geometry variant information is given for each result).

### 4.3. 3D Hirshfeld Surfaces (3D HS)

The 3D Hirshfeld surfaces (3D HS) technique was used to investigate intermolecular interaction patterns in solid state [31,32]. The outer contour of the space of a molecule in a crystalline environment is referred to as the 3D HS. It is constructed using a quotient of the promolecule and the electron density of the procrystal. The d_norm_, shape index, and curvedness surfaces projected over the 3D HS reveal hydrogen bonds and van der Waals contacts [32,90]. The 3D HS can be decomposed into a 2D ‘molecular fingerprint’ (2D FP) map, which is a plot of the distances of each surface point to the neighboring interior and exterior atoms (d_i_ versus d_e_). It describes the distribution of the molecule interactions with its environment. The calculations were carried out using Gaussian 16, rev. C01 [81], and CrystalExplorer [31,32,90].

The enrichment ratio, E_XY_, a descriptor derived from 2D FP [91], is defined as the ratio of the proportion of actual connections in a crystal to the theoretical fraction of random interactions. It denotes a tendency to establish or refrain from interactions [91]. To speed up this task, a Python 3.10.11 script was written.

### 4.4. Molecular Docking

The molecular docking (MD) technique requires a reliable ligand structure. Three-dimensional (3D) molecular structures of the inosine, guanosine, and acadesine have been optimized using Gaussian 16, rev. C01 [81], at the M062X/6-31+G(d,p) [82] level of theory.

The IR frequency criterion was used to validate the geometry. A search was conducted for the presence of imaginary infrared frequencies. The presence of such frequencies would indicate the presence of saddle points on the potential energy surface, as well as evidence of molecular instability.

Structures of the targets and complexes used for the conformational studies were retrieved from the Protein Data Bank PDB database (http://www.rcsb.org/pdb; 19 November 2024).

The files with receptor and ligand structures were converted to the .pdbqt format using MGLTools ver. 1.5.7. Molecular docking was performed using automated docking tools AutoDock ver. 4.2.6 [92] and AutoDock Vina ver. 1.2.3 [93]. Prior to docking the ligands, the native ligand that co-crystallized with the RdRp and water molecules were removed from the structure and the protonation state of the protein was corrected. A redocking task was performed to evaluate the efficacy of the docking process. It was considered successful when the root mean square deviation of the atomic position of a pose relative to its native structure did not exceed 3 Å. Six studied ligands were incorporated into RNA primer, combined with RNA template, and then docked into the binding site in RdRp.

Docking into the protein pocket was performed using a template and a defined search space around the active site. At the first step, the preliminary grid box of size 9–15 Å centered on the active site was defined. Following the docking process, the grid box size was adjusted. The optimal poses that resulted in the stabilization of the protein–ligand complex with the highest docking score were selected for further investigation. The docking results were verified using a genetic evolutionary method for molecular docking (GEMDOCK) [94]. This approach incorporates a distinct evolution operator alongside the utilization of a genetic algorithm and scoring function using a piecewise potential energy function. The energy terms’ separation is based on GEMDOCK [94] with the use of a task automation script written by us in Python 3.10.11. The Gehlhaar model [95] with original empirical parameterization and PRODIGY approach [96] were used for the estimation of the binding affinity. The final 2D and 3D visualizations of the binding modes were made using VMD [97]. This combination of techniques has proven effective in our previous studies.

### 4.5. Root Mean Square Deviation of the Binding Modes

The average deviation between the binding modes was calculated using the newly defined quantity: root mean square deviation of the binding modes (RMSD_BM) [1]. It was calculated as follows:

(14) $$RMSD\_BM(P,Q)=\sqrt{\frac{1}{n}\sum_{i}{\left(p_{i}-q_{i}\right)}^{2}}$$

where p_i_ and q_i_ are the binding interactions in each structure and P = {p_i_} and Q = {q_i_}.

### 4.6. Structure-Binding Strength Indices

To assess differences in the protein–ligand binding affinity, we used the set of new parameters, co-called structure-binding strength indices (SBSI) [98]. A common mathematical form is shared by all of them. The first five indices were defined in our previous papers [98,99], but the last two are completely new:

(a) Structure—Binding Affinity Index, SBAI:
  (15) $$SBAI=\frac{{BA}_{reference}-{BA}_{ligand}}{1-s}=\frac{d_{BA}}{1-s}$$
  which measures the difference in the protein–ligand binding affinity between the candidate and reference ligands (numerator) with respect to their structural similarity (denominator);
(b) Structure—Hydrogen Bonds Index, SHBI:
  (16) $$SHBI=\frac{{HB}_{reference}-{HB}_{ligand}}{1-s}=\frac{d_{HB}}{1-s}$$
  which measure difference in the energy of hydrogen bonds (connecting the protein with the ligand) between the complex with the selected ligand and the reference compound (numerator) with respect to their structural dissimilarity, 1 − s, (denominator);
(c) Structure—Steric Effect Index, SSEI:
  (17) $$SSEI=\frac{{SE}_{reference}-{SE}_{ligand}}{1-s}=\frac{d_{SE}}{1-s}$$
  which measures difference in the energy of steric effects between the complex with the selected ligand and the reference (numerator) with respect to their structural dissimilarity, 1 − s, (denominator);
(d) Structure—Protein-Ligand Index, SPLI:
  (18) $$SPLI=\frac{{PL}_{reference}-{PL}_{ligand}}{1-s}=\frac{d_{PL}}{1-s}$$
  which measures the difference in the strength of the protein–ligand interactions between the ligand and reference (numerator) with respect to their structural dissimilarity, 1 − s, (denominator);
(e) Structure—van der Waals Index, SVdWI:
  (19) $$SVdWI=\frac{{VdW}_{reference}-{VdW}_{ligand}}{1-s}=\frac{d_{VdW}}{1-s}$$
  which measures the difference in the van der Waals term in the presence of the ligand and reference (numerator) with respect to the structural dissimilarity, 1 − s, (denominator);
(f) Structure—RNA index, SRNAI:
  (20) $$SRNAI=\frac{{RNA}_{reference}-{RNA}_{ligand}}{1-s}=\frac{d_{RNA}}{1-s}$$
  which measures the difference in the strength of the interactions between the RNA template and ligand, and the RNA template and reference (numerator) with respect to their structural dissimilarity, 1 − s, (denominator);
(g) Structure—Metal Index, SMEI:
  (21) $$SMEI=\frac{{ME}_{reference}-{ME}_{ligand}}{1-s}=\frac{d_{ME}}{1-s}$$
  which measures the difference in the strength of the interactions between the RNA template and ligand, and the RNA template and reference (numerator) with respect to their structural dissimilarity, 1 − s, (denominator);

The d_i,_ i∈{BA, HB, SE, PL, VdW, RNA, ME} in the numerator is derived from the molecular docking results, while s in the denominator is the molecular similarity. In this paper, Tanimoto, atom pair Tanimoto, and maximum common substructure were used to describe structural similarity, s. They were calculated using classical definitions and our Python script (validated on the set of compounds). Among them, the maximum common substructure (graph-theory-based) is the most sensitive to structural differences.

The mathematical foundation of the SBSI formalism can be found in our previous papers [1,98,99]. The calculations were performed by means of proprietary software developed within Visual Studio 2022, C#. The purpose of this approach is to expedite the computational processes.

### 4.7. Molecular Dynamics Simulation (MSD)

The molecular dynamics simulations have been performed using the coarse grain technique [96,100], which reduces computational complexity by replacing the real particles with a smaller number of representative elements. Their behavior is equivalent to the original ones but can be easily tracked during the simulation. This method facilitates the docking of large biological complexes within a reasonable time frame. However, its performance depends on the number of models generated at each step. The total number of generated models was 50,000, 2% of which were selected, and the top 10 were subject to further analysis. The simulated temperature is 300 K, typical for biological compounds. (The dynamics of the complex at native temperature was also under investigation, but these results are not meaningful.) The Amber-derived forcefield implemented in HADDOCK was used. The root mean square fluctuation (RMSF), defined as the time average of the root mean square deviation (RMSD) of atomic positions, was analyzed regarding the flexibility of individual regions. The degree of variability of the radius of gyration was investigated (to speed up this task, a Python 3.10.11 script was written).

All of the graphs, data analysis, and 3D visualizations were made using OriginPro v. 10.1 (2024) and VMD 1.9.3 [97].

## 5. Conclusions

The present study focuses on the analysis of a commercially available sample of the broad-spectrum antiviral ribavirin. Ribavirin exhibits structural similarity to 1*H*-1,2,4-triazole or acadesine, yet in terms of biological activity, it is more akin to favipiravir.

Due to the high relaxation coefficients, RBV is a difficult material to study experimentally, and our attempts to detect the signal using a pulse spectrometer have been unsuccessful. Moreover, from a measurement perspective, the absence of protons at the N(2) and N(4) positions is an additional problematic issue. The ^1^H-^14^N double resonance technique used in this study is in fact a combination of the classical cross-relaxation technique and a new cross-polarization technique designed particularly for the fast field-cycling (FFC) relaxometers. The implementation of this technique facilitated the retrieval of the complete spectrum, thereby encompassing all 12 resonance lines. A reliable interpretation of such a complex spectrum is not possible solely based on comparison with other compounds and required the use of solid-state GGA methods. Based on the PXRD pattern, ^1^H-^14^N cross-relaxation spectra, and GGA/RPBE modelling, the presence of the polymorphic form II in the studied material has been ascertained. The distinction between both forms of RBV is readily apparent.

Following a thorough analysis of the binding mode, it was determined that there are some common features that are consistent in both the solid state and protein–ligand complex. The present study identified the intramolecular hydrogen bond NH···N in RBV as playing a crucial role in the formation of a quasi-five-membered ring. However, this bond was found to be inadequate for the positioning of the amide group in the ring plane. In form I of RBV, the amide group participates in strong NH⋯O and OH⋯O hydrogen bonds, whereas in form II, it is involved in strong NH⋯N hydrogen bonds, weaker NH⋯O hydrogen bonds, and weak NH⋯HC dispersive contacts. In form II, unlike form I, glycones bind together by OH⋯O bonds. In contrast, the basic binding motifs in protein–ligand complexes of RBV are strong NH⋯N and NH⋯O hydrogen bonds, while glycone moieties do not bind to each other. From the standpoint of biological activity, the capacity of the amide group of the aglycone to form hydrogen bonds appears to be of greater significance than its potential to bind the three hydroxyl groups of the glycone. The key role in ligand binding in the complex is played by the amide group and glycone, which explains why replacing pyridazine with triazole does not significantly affect the binding affinity. The glycone is a vital component in the incorporation of RBV into the RNA primer, while the amide group of the aglycone serves to bind the RNA primer to the RNA template. The high-anti conformation of the ribofuranosyl moiety in the stable form II of RBV is different from the anti conformation adopted by RBV, RBV-MP, or RBV-TP in protein–ligand complexes. The only exception is RdRp *Murine norovirus*, which also incorporates high-anti. Nevertheless, the anti-adopted protein–ligand complex exhibits a higher degree of similarity in its conformation to form II than form I. The replacement of the carbon adjacent to the ribose with nitrogen, in conjunction with the absence of oxygen at the 2-position of the ring, results in an increased flexibility of the RBV in comparison to the favipiravir-ribofuranosyl. The ribofuranosyl in RBV has been demonstrated to inhibit tautomerism and to freeze the conformation of the amide group. The results of the molecular dynamics simulations suggests that RBV and favipiravir-ribofuranosyl incorporated into the RNA primer exhibit comparable stability within the protein binding region.

Although acadesine is the most similar in terms of binding mode to RBV, its binding capacity is weaker than RBV, while the much more divergent T-705 and T-1106 bind more strongly. The formation of significantly stronger hydrogen bonds between the RNA primer with incorporated T-705 or T-1106 as ligand and the RNA template seems responsible for this effect. The titular anti-butterfly (inverted butterfly) effect is associated with the consequences of both the aglycone moiety and the neighborhood alteration. As shown, the RBV binding abilities make it much more similar to T-705 or T1106 than to the structurally related acadesine, guanosine, and inosine.

The seven structure-binding strength indices and six novel quadrupolar indices defined in this study have been proven to facilitate the evaluation of the similarity of binding motifs in the solid state and protein–ligand complex. They assess the degree to which binding motifs present in the solid state are realized in protein–ligand complexes. The propensity of nitrogen atoms to form a particular bond is marginally modified during the transition from a solid state to a complex state. Furthermore, the RBV nitrogen atoms that exhibit strong binding in the solid state are highly susceptible to participation in strong bonds in protein–ligand complex.

The ^1^H-^14^N NQR cross-relaxation spectra in conjunction with XPRD pattern, in-depth crystallographic data analysis, molecular docking, and molecular dynamics simulation revealed ribavirin’s ability to form specific intermolecular bonds in solid state and protein–ligand complex. This renders ribavirin analogous, in terms of binding mode and action, to favipiravir. Such a combined approach is helpful in predicting and validating the high-affinity drug binding mode. In consideration of the findings, the compounds under investigation were categorized into two classes, a classification that corresponds to a division based on their biological activity scope.

In this paper, we presented a method for screening active and inactive compounds, which is based on new parameters and a new technique. Identification of effective inhibitors represents a significant challenge in the field of antiviral research. This finding indicates that the proposed method functions effectively in a practical setting as a screening protocol.

In conclusion, the combined approach proposed in this paper has the capacity to introduce a novel universal paradigm to the field of drug design.

## Figures and Tables

**Table 1:** A comparison of the biological activity profiles and structural similarity parameters (Tanimoto, atom pair Tanimoto, and maximum common substructure) of ribavirin and its analogues shown in Figure 2.

Compound	CAS	Biological Activity Profile	Similarity		
			Tanimoto *	Atom Pair Tanimoto **	Maximum Common Substructure ***
Ribavirin, 1-(β-D-Ribofuranosyl)-1*H*-1,2,4-triazole-3-carboxamide, RBV	36791-04-5	broad spectrum anti-viral, potential anti-cancer	1.0	1.0	1.0
Guanosine, 9-(β-D-Ribofuranosyl)guaninę, G	118-00-3	required for an RNA splicing reaction in mRNA; neuroprotective agent reducing neuroinflammation, oxidative stress, and excitotoxicity	0.4476	0.598	0.5417
Inosine, 9-(β-D-Ribofuranosyl) hypoxanthine, I	58-63-9	regulation of RNA editing, metabolic enzyme activity, and signaling pathways; promotion of T cell proliferation and differentiation and enhancement of the response to immune-checkpoint inhibitors therapy; anti-inflammatory, antinociceptive, immunomodulatory, and neuroprotective	0.4526	0.560	0.5652
Acadesine, 5-aminoimidazole-4-carboxamide-1-β-d-ribofuranoside, AICA-riboside/AICAR	2627-69-2	hypoglycemic, cardioprotective, and anti-neoplastic agent; regulates lipid and glucose metabolism, pro-inflammatory responses, cytokine production, cell proliferation, and apoptosis; improve ischemia or reperfusion injury and kidney fibrosis in rats; protection against acute tubular necrosis; potential anti-cancer	0.4675	0.731	0.8421
Favipiravir -ribofuranosyl, 6-fluoro-3-hydroxy-2-pyrazinecarboxamide-ribofuranosyl, Favipiravir -Ry, FVP-R	740790-94-7	broad spectrum anti-viral, potential anti-cancer	0.4342	0.545	0.4231
Favipiravir -ribofuranosyl (F-deprived), 4-[(2*R*,3*R*,4*S*,5*R*)-3,4-dihydroxy-5-(hydroxymethyl)oxolan-2-yl]-3-oxopyrazine-2-carboxamide	356782-84-8	yellow fever virus anti-viral	0.4298	0.615	0.4400

* the Tanimoto similarity between two sets, ** the topological distance between atom pairs, *** the maximal common edge subgraph.

**Table 2:** ^14^N NQR frequencies, quadrupole coupling constants e^2^qQ/h, and asymmetry parameters η for RBV determined from ^1^H-^14^N NQR cross-relaxation spectra detected at T = 295 K.

Nitrogen Site	ν_+_ (MHz)	ν_−_ (MHz)	ν_0_ (MHz)	|e^2^qQ/h| (MHz)	η	Assignment
N(1)	2.340	1.700	0.640	2.693	0.475	–NH_2_
N(2)	3.980	2.650	1.330	4.420	0.602	–N= ring position N(2)
N(3)	2.600	2.280	0.320	3.253	0.197	–N= ring position N(4)
N(4)	2.400	1.480	0.920	2.587	0.711	>N-sugar

**Table 3:** ^14^N NQR frequencies, quadrupole coupling constants e^2^qQ/h, and the asymmetry parameters η in 1*H*-1,2,4-triazole at RT [44] and 77K (in parentheses) [45,46].

Nitrogen Site	ν_+_ (MHz)	ν_−_ (MHz)	ν_0_ (MHz)	|e^2^qQ/h| (MHz)	η	Assignment
1*H*-1,2,4-triazole	3.793 (3.877)	2.405 (2.470)	1.384 (1.407)	4.132 (4.231)	0.672 (0.665)	–N= ring position N(2)
	2.503 (2.551)	2.257 (2.279)	0.246 (0.272)	3.173 (3.220)	0.155 (0.169)	–N= ring position N(4)
	2.114	1.442	0.672	2.371	0.567	>NH

**Table 4:** ^14^N NQR frequencies, quadrupole coupling constants e^2^qQ/h, and the asymmetry parameters η for RBV (forms I and II) calculated at GGA/RBPE level of theory.

Polymorph	Structure Geometry	ν_+_ (MHz)	ν_−_ (MHz)	ν_0_ (MHz)	|e^2^qQ/h| (MHz)	η	Assignment
RBV form II	hydrogen positions optimized	2.503	1.755	0.727	2.629	0.750	–NH_2_
		4.107	2.663	1.444	4.513	0.640	–N= ring position N(2)
		2.655	2.619	0.035	3.516	0.020	–N= ring position N(4)
		2.465	1.479	0.986	2.852	0.511	>N-sugar
RBV form II	hydrogen positions optimized	2.491	2.441	0.049	3.288	0.030	-NH_2_
		3.878	2.043	1.835	3.947	0.930	–N= ring position N(2)
		2.686	2.096	0.590	3.188	0.370	–N= ring position N(4)
		2.397	1.338	1.058	2.490	0.851	>N-sugar
RBV form I	full geometry optimization	2.490	1.828	0.662	2.879	0.460	–NH_2_
		4.004	2.578	1.426	4.388	0.650	–N= ring position N(2)
		2.629	2.612	0.017	3.494	0.010	–N= ring position N(4)
		2.401	1.514	0.887	2.610	0.680	>N-sugar
RBV form I	full geometry optimization	2.593	2.165	0.428	3.172	0.270	–NH_2_
		3.694	1.931	1.763	3.750	0.940	–N= ring position N(2)
		2.579	2.512	0.068	3.394	0.040	–N= ring position N(4)
		2.402	1.462	0.940	2.576	0.730	>N-sugar

**Table 5:** The <C(5)N(1)CO conformation angle for ribavirin and its analogues (X-ray structures from [41,50,51,52,53,54]).

Structure	<C(5)N(1)CO
Ribavirin, form I	10.41
Ribavirin, form II	119.00
Favipiravir -ribofuranosyl, Favipiravir—ribofuranosyl, 3,4-dihydro-6-fluoro-3-oxo-4-β-D-ribofuranosyl-2-pyrazine carboxamide, T-705	15.40
3,4-dihydro-3-oxo-4-β-D-ribofuranosyl-2-pyrazine carboxamide, T-1106	7.62
Acadesine	62.77
Inosine, α-form	12.44
Inosine, β-form	139.87
Guanosine.H_2_O	122.49

**Table 6:** The Euclidean distance (ED) and root mean square deviation (RMSD) calculated between the 3D HS percentage contributions for ribavirin form II and other compounds studied (X-ray structures from [41,50,51,52,53,55,56,57,58,59,60,61,62,63]).

Structure	ED	RMSD
Ribavirin, form I, 295 K *	18.7	4.83
1*H*-1,2,4-triazole, 15 K **	58.2	15.03
1*H*-1,2,4-triazole, 113 K	59.4	15.33
1*H*-1,2,4-triazole, 120 K	58.0	14.99
1*H*-1,2,4-triazole, 295 K	60.1	15.51
1*H*-1,2,4-triazole-3-carboxamide, 298 K	29.9	7.73
Favipiravir-ribofuranosyl, 3,4-dihydro-6-fluoro-3-oxo-4-β-D-ribofuranosyl-2-pyrazine carboxamide, T-705, 100 K	17.6	4.55
3,4-dihydro-3-oxo-4-β-D-ribofuranosyl-2-pyrazine carboxamide, T-1106, 100 K	13.2	3.41
Favipiravir, 100 K **, form I	37.8	9.75
Favipiravir, 293 K, form I	36.9	9.52
Favipiravir, 296 K, form I	36.9	9.52
Favipiravir, 298 K, form II	34.2	8.83
Acadesine	55.4	14.31
Inosine, α-form	49.9	12.89
Inosine, β-form	51.3	13.26

* Ribavirin form II is a reference; ** the value averaged over available structures.

**Table 7:** Enrichment ratios E_XY_ characterizing the various contacts in RBV and its analogues (X-ray structures from [41,50,51,52,53,54]).

Structure	Atom	C	H	N	O	F
Ribavirin, form I	Surface %	5.15	65.3	11.00	18.55	-
	C	2.16	0.00	-	-	-
	H	0.92	0.76	0.90	-	-
	N	2.84	0.00	1.38	0.17	-
	O	0.05	2.72	1.18	0.44	0.20
Ribavirin, form II (stable)	Surface %	4.30	62.25	11.45	22.00	-
	C	2.16	-	-	-	-
	H	0.92	0.82	-	-	-
	N	2.84	0.90	2.52	-	-
	O	0.05	1.58	0.12	0.00	-
T-705	Surface %	7.5	57.3	6.6	20.25	8.45
	C	0.00	-	-	-	-
	H	0.90	0.85	-	-	-
	N	0.00	1.36	0.00	-	-
	O	1.15	1.35	0.67	0.17	-
	F	3.00	0.97	0.99	0.70	0.03
T-1106	Surface %	7.95	61.6	6.4	24.05	-
	C	0.00	-	-	-	-
	H	0.81	0.92	-	-	-
	N	0.00	1.23	0.00	-	-
	O	2.09	1.20	1.01	0.12	-
Favipiravir, form I	Surface %	13.45	39.2	16.45	17.05	14.05
	C	0.94	-	-	-	-
	H	0.77	0.89	-	-	-
	N	1.36	1.01	1.52	-	-
	O	1.61	1.41	0.77	0.10	-
	F	0.53	1.02	0.30	0.61	1.11
Favipiravir, form II	Surface %	13.05	39.7	15.9	17.2	14.25
	C	0.00	-	-	-	-
	H	0.92	0.81	-	-	-
	N	1.95	0.90	1.50	-	-
	O	1.56	1.34	0.84	0.00	-
	F	0.40	1.32	0.04	0.92	0.75
	Surface %	6.1	70.0	7.35	16.65	-
Acadesine	C	0.00	-	-	-	-
	H	1.28	0.86	-	-	-
	N	0.56	1.30	0.56	-	-
	O	0.39	1.37	0.08	0.07	-
	Surface %	7.35	60.6	11.75	20.2	-
	C	1.11	-	-	-	-
Inosine, α-form	H	0.85	0.84	-	-	-
	N	1.10	1.28	0.43	-	-
	O	1.35	1.36	0.46	0.10	-
Inosine, β-form (stable)	Surface %	8.45	63.45	9.7	18.4	-
	C	3.78	-	-	-	-
	H	0.40	1.04	-	-	-
	N	1.89	0.80	1.91	-	-
	O	1.32	1.24	0.81	0.12	-

**Table 8:** The Euclidean distance (ED) between the enrichment ratios for both forms of RBV and related compounds.

Structure	ED	
	RBV Form II	RBV Form I
RBV	5.09	5.09
T-705	5.68	3.74
T-1106	4.93	0.88
Favipiravir, form I	3.26	3.02
Favipiravir, form II	3.56	3.27
Acadesine	3.76	2.52
Inosine, α-form	3.24	2.11
Inosine, β-form	2.54	4.85

**Table 9:** The percentage contributions to the local 3D Hirshfeld surface at all nitrogen sites in a molecule and the root mean square deviation (RMSD_N) between the 3D HS percentage contributions for ribavirin form II and other compounds studied.

	N···C	N···H	N···N	N···O	N···F	RMSD_N
Ribavirin, form I	38.1	60.3	0	1.6	-	4.86
Ribavirin, form II	43.6	51.5	3.2	1.8	-	0 *
T-705	45.3	51.4	0	1.7	1.6	1.77
T-1106	45.3	52.7	0	2.0	-	1.71
Favipiravir, form I	43.2	48.2	3.1	3.0	2.4	1.91
Favipiravir, form II	44.5	47.1	3.1	3.2	2.1	2.30
1*H*-1,2,4-triazole	39.5	39.2	21.3	-	-	9.96
1*H*-1,2,4-triazole-3-carboxamide	33.2	63.7	3.2	-	-	7.17
Inosine, α-form	56.6	42.7	0.3	0.4	-	7.17
Inosine, β-form	59.4	33.3	0.5	6.8	-	11.07
Acadesine	39.4	59.7	0.2	0.7	-	4.36
Guanosine.H_2_O	50.8	48.2	0.7	0.3	-	3.77

* reference.

**Table 10:** The total interaction energy partitioned into electrostatic, polarization, dispersion, and repulsion terms (in kJ/mol), summarized over molecular clusters of 10 Å in diameter.

Structure	E_electrostatic_	E_polarization_	E_dispersion_	E_repulsion_	E_total_
Ribavirin, form I	−205.58	−46.75	−138.88	140.47	−250.79
Ribavirin, form II	−253.46	−50.71	−125.55	168.60	−261.28
T-705	−196.01	−42.99	−117.32	140.83	−215.56
T-1106	−166.51	−40.04	−133.96	129.24	−211.35
Favipiravir, form I	−79.49	−14.81	−107.71	62.57	−139.47
Favipiravir, form II	−61.76	−11.85	−67.63	48.19	−93.08
1*H*-1,2,4-triazole	−93.36	−17.12	−51.01	63.23	−98.28
1*H*-1,2,4-triazole-3-carboxamide	−164.93	−37.25	−87.89	106.25	−183.91
Inosine, β-form	−204.66	−41.24	−132.37	153.85	−224.51
Acadesine	−190.61	−40.59	−129.09	135.91	−224.45

**Table 11:** The hydrogen bonds within (**a**) form I and (**b**) form II of RBV in solid state.

Polymorph	Moiety	Type of Interactions	Hydrogen Bond	d_DA_ [Å]	<DAH
RBV, Form I	aglycone	intramolecular	NH···N(2)	2.793	81.40
		intermolecular	NH···O	3.251	143.92
			NH···O	2.976	156.63
			O···HO	2.685	154.94
			O···HC	3.464	162.63
			N(4) ···HO	2.860	153.48
			N(2) ···HO	2.899	138.27
RBV, Form II	aglycone	intramolecular	NH···N(2)	2.806	94.46
		intermolecular	NH···N(4)	2.940	167.00
			NH…O	3.242	110.98
			NH···HC	4.152	153.72
			O···HO	2.689	156.39
			C(5) ···HO	3.283	176.19
	glycone		C···HO	3.456	147.84
			O···HO	2.684	159.04
			O···HO	2.745	158.23

**Table 12:** The binding of the ligand RBV-MP or FVP-RMP with RdRp protein (7DFH and 7DFG, respectively).

Ligand	Interaction Type	Residue	H-A	D-A	<DAH	Donor	Acceptor
RBV	Hydrogen bond	Arg555	3.02	3.40	104.28	N	O
		Asp623	2.58	3.06	110.83	O	N3
		Asp623	3.39	3.97	121.64	O	O
		Ser682	3.24	4.07	142.89	–NH from amine	O3
		Ser682	3.12	3.47	102.94	–N(2)=	O2
	Metal complexation	Mg^2+^	-	2.72	-	RBV-MP	P
FVP	Hydrogen bond	Lys545	2.86	131.57	131.57	–NH_2_	N3
		Lys545	3.09	3.64	114.95	–N(3)	N2
		Asp623	3.50	3.97	111.96	–NH from amine	O3
		Thr680	3.11	3.87	135.50	O3	O3
	Metal complexation	Mg^2+^	-	2.84	-	FVP-RMP	P

**Table 13:** The conformations of the glycone moiety and the dihedral angles between rings in structures containing RBV moiety as a ligand.

Structure	Protein	Type	Ligand	χ [°] <C(5)NCO	χ’ [°] <N(2)NCO	θ [°] Ring Angle	Conformation	Helix Type	Binding Affinity [kJ/mol]
7DFG [67]	SARS-CoV-2, RdRp	positive-single stranded RNA	FVP-RMP	32.0	−148.5	36.9	anti	C3′-endo sugars (A-helix)	−71.98 * (−22.91 **)
7DFH [68]	SARS-CoV-2, RdRp	positive-single stranded RNA	RBV-MP	40.8	−139.6	41.0	anti		-50.34 (−19.11 **)
8KI6 [71]	Tomato spotted wilt virus L, RdRp	negative-stranded RNA	RBV	75.5	−104.5	78.9	anti		−16.07
3SFU [72]	Murine norovirus, RdRp	positive-single-stranded RNA	RBV	97.0	−84.6	70.5	high-anti		−16.85
1ME8 [73]	Inosine-5′-monophosphate dehydrogenase	enzyme, DNA/RNA synthesis	RBV-MP	84.2	−95.7	79.3	anti		−26.07
1NF7 [74]	Inosine-5′-monophosphate dehydrogenase	enzyme, DNA/RNA synthesis	RBV-MP	81.0	−99.2	78.1	anti		−26.07
[41]	Solid	Form II	RBV	119	−62.2	78.2	high-anti		-
-	Solid, optimized ***	Form II	RBV	122.5	−61.7	78.0	high-anti		-
[41]	Solid	Form I	RBV	10.4	−170.6	77.7	anti		-
-	Solid optimized ***	Form I	RBV	13.46	−171.7	77.9	anti		-
[50]	Solid	-	FVP-R	15.4	−164.9	83.9	anti		-
5AXD [75]	Mouse S-adenosyl homocysteine hydrolase, SAHH	DNA/RNA metabolism	RBV	75.3	−107.5	76.3	anti	C3′-endo sugars (A-helix)	−24.83
1R6A [76]	Denge virus, 2′-o-methyltransferase	positive-single-stranded RNA	RBV-MP	41.0	−138.9	89.1	anti		−22.62
2E9R [77]	Foot-and-mouth disease (FMDV) virus, RdRp	positive-single-stranded RNA	RBV-TP	−11.9	167.5	71.0	anti		−22.09

* the ligand incorporated into primer; ** the ligand itself; *** full geometry optimization (unit cell fixed).

**Table 14:** The docking results for studied ligands. The energy of protein–ligand, steric, van der Waals, and hydrogen bond interactions is expressed in kJ/mol.

Parameter	T-705	T-1106	RBV	Inosine	Acadesine	Guanosine
Total energy	−498.80	−496.06	−448.26	−402.65	−402.98	−383.74
Protein–ligand	−191.67	−188.93	−207.44	−200.96	−203.64	−177.36
Steric	−134.72	−135.66	−155.14	−150.54	−156.29	−138.14
Hydrogen bonds	−21.75	−18.06	−19.28	−16.50	−14.32	−6.19
VdW	−55.00	−55.00	−60.55	−44.85	−50.66	−60.69
Mg^2+^ Zn^2+^	−40.47	−40.47	−42.95	−42.95	−42.95	−42.95
RNA template	−292.90	−266.65	−197.87	−202.59	−199.08	−206.89

**Table 15:** The comparison of the binding modes using mathematical metrics.

Ligand	RMSD_BM (kJ/mol)	Manhattan Distance (kJ/mol)
T-705	5.742	276.32
T-1106	5.747	278.89
Inosine	5.934	427.14
Acadesine	2.864	184.35
Guanosine	6.120	456.79

**Table 16:** The bonding affinity (in kJ/mol) and structure binding affinity indices (SBAI) calculated on the assumption of different similarity factors (listed in Table 1).

Ligand	Binding Affinity (kJ/mol)	SBAI (kJ/mol)		
		Tanimoto Similarity	Atom Pair Tanimoto Similarity	Maximum Common Substructure Similarity
T-705	−71.98	38.2	37.5	47.6
T-1106	−68.51	31.9	32.4	47.2
RBV	−50.34	100.0	100.0	100.0
Inosine	−31.43	−34.5	−43.5	−43.0
Acadesine	−29.84	−38.5	−129.8	−76.2
Guanosine	−27.13	−42.0	−50.6	−57.7

**Table 17:** The solid-state effect in both forms of RBV calculated at GGA/RBPE level of theory.

Form	Solid State		Single Molecule		Solid-State Effect		Assignment
	|e^2^qQ/h| (MHz)	η	|e^2^qQ/h| (MHz)	η	Δ^s^ (MHz)	δ^s^	
RBV I	3.288	0.030	3.946	0.110	−0.658	−0.08	-NH_2_
	3.947	0.930	4.142	0.680	−0.195	0.25	–N= ring position N(2)
	3.188	0.370	3.926	0.090	−0.738	0.28	–N= ring position N(4)
	2.490	0.851	2.649	0.730	−0.159	0.121	>N-sugar
RBV II	2.629	0.750	3.758	0.070	−1.129	0.68	–NH_2_
	4.513	0.640	4.527	0.660	−0.014	−0.02	–N= ring position N(2)
	3.516	0.020	4.063	0.130	−0.547	−0.11	–N= ring position N(4)
	2.852	0.511	2.734	0.670	0.118	−0.159	>N-sugar
FVP I	2.336	0.81	2.968	0.51	−0.632	0.3	–NH_2_
	4.56	0.24	4.829	0.32	−0.269	−0.08	–N= ring position N(4)
	4.739	0.35	4.564	0.33	0.175	0.02	–N= ring position N(1)
FVP II	2.308	0.76	2.935	0.48	−0.627	0.28	–NH_2_
	4.615	0.34	4.839	0.4	−0.224	−0.06	–N= ring position N(4)
	4.676	0.38	4.554	0.39	0.122	−0.01	–N= ring position N(1)

**Table 18:** The protein–ligand complexation effect calculated at GGA/RBPE level of theory.

	Protein-Ligand Complex		Single Molecule		Complexation Effect		Assignment
	|e^2^qQ/h| (MHz)	η	|e^2^qQ/h| (MHz)	η	Δ^c^ (MHz)	δ^c^	
RBV	2.742	0.610	4.212	0.230	−1.47	0.38	–NH_2_
	4.810	0.630	4.662	0.650	0.148	−0.02	–N= ring position N(2)
	4.519	0.090	4.525	0.070	−0.006	0.02	–N= ring position N(4)
	2.842	0.511	2.850	0.640	−0.008	−0.129	>N-sugar
FVP-R	2.445	0.300	3.810	0.120	−1.365	0.18	–NH_2_
	4.637	0.420	4.784	0.400	−0.147	0.02	–N= ring position N(4)
	1.989	0.180	2.283	0.400	−0.294	−0.22	–N= ring position N(1)

**Table 19:** The solid-state versus protein–ligand complexation effect in RBV calculated at GGA/RBPE level of theory.

	Form II		Form I		Assignment
	Δ^CS^ (MHz)	δ^cs^	Δ^CS^ (MHz)	δ^CS^	
RBV	0.113	−0.14	−0.546	0.58	–NH_2_
	0.297	−0.01	0.863	−0.3	–N= ring position N(2)
	1.003	0.07	1.331	−0.28	–N= ring position N(4)
	−0.01	0	0.352	−0.34	>N-sugar
FVP/FVP-R	−0.109	0.51	−0.137	0.46	–NH_2_
	−0.077	−0.18	−0.022	−0.08	–N= ring position N(4)
	2.75	0.17	2.687	0.2	–N= ring position N(1)/>N-sugar

## Data Availability

All data are included in the text of the manuscript.

## Abbreviations

3D HS	3D Hirshfeld surfaces
2D FP	2D molecular fingerprints
CAS	Chemical abstracts service
CR	Cross-relaxation
DFT	Density functional theory
ED	Euclidean distance
EM	Electron microscopy
E_XY_	Enrichment ratios
FFC	Fast field-cycling
FVP	Favipiravir
GGA	Generalized gradient approximation
IMP	Inosine monophosphate
IMPDH	Inosine-5′-monophosphate dehydrogenase
M06L, M062X	Meta-hybrid Minnesota functionals
MD	Molecular docking
MDS	Molecular dynamic simulations
mRNA	Messenger RNA
NAD+	Nicotinamide adenine dinucleotide
NMR	Nuclear magnetic resonance
NQR	Nuclear quadrupole resonance
PXRD	Powder X-ray diffraction
QTAIM	Quantum theory of atoms in molecules
QI	Quadrupolar indices
RBV	Ribavirin
RdRp	RNA-dependent RNA polymerase
RDS	Reduced density gradient
RMDS	Root mean square distance
RMSD	BM root mean square deviation of the binding modes
RMP	Ribavirin 5-monophosphate
RMSF	Root mean square fluctuation
RNA	Ribonucleic acid
RPBE	Revised Perdew–Burke–Ernzerhof
RTP	Ribavirin 5-triphosphate
SARS-CoV-2	Severe acute respiratory syndrome coronavirus 2
SBAI	Structure—binding affinity index
SBSI	Structure—binding strength indices
SHBI	Structure—hydrogen bonds index
SMEI	Structure—metal index
SSEI	Structure—steric effect index
SPLI	Structure—protein–ligand index
SRNAI	Structure—RNA index
SVdWI	Structure—van der Waals index
T-705	3,4-dihydro-6-fluoro-3-oxo-4-β-D-ribofuranosyl-2-pyrazine carboxamide
T-1106	3,4-dihydro-3-oxo-4-β-D-ribofuranosyl-2-pyrazine carboxamide
TK	Tkatchenko–Scheffler method
XMP	Xanthosine monophosphate (XMP)
